# Integrated Analysis of Transcriptome and Proteome to Reveal Pupal Color Switch in *Papilio xuthus* Butterflies

**DOI:** 10.3389/fgene.2021.795115

**Published:** 2022-02-03

**Authors:** Jin-Wu He, Zhi-Wei Dong, Ping Hu, Wei Liu, Ru Zhang, Gui-Chun Liu, Ruo-Ping Zhao, Wen-Ting Wan, Wen Wang, Xue-Yan Li

**Affiliations:** ^1^ State Key Laboratory of Genetic Resources and Evolution, Kunming Institute of Zoology, Chinese Academy of Sciences, Kunming, China; ^2^ Kunming College of Life Science, University of Chinese Academy of Sciences, Kunming, China; ^3^ School of Ecology and Environment, Northwestern Polytechnical University, Xi’an, China

**Keywords:** pupal color polyphenism, PCMH, *Papilio xuthus*, neuroendocrine system, toll signaling pathway, juvenile hormone related genes, cuticular proteins

## Abstract

Pupal color polyphenism in *Papilio* butterflies, including green, intermediate, or brown, is an excellent study system for understanding phenotypic plasticity. Previous studies suggested that development of brown pupae may be controlled by a hormone called pupal-cuticle-melanizing-hormone (PCMH) which is synthesized and secreted from brain-suboesophageal ganglion and prothoracic ganglion complexes (Br-SG-TG1) during the pre-pupa stage. However, detailed molecular mechanisms of neuroendocrine regulation in pupal color development remain unknown. In this study, we integrated the expression profiles of transcriptome and proteome at pre-pupa stages [2 h after gut purge (T1) and 3 h after forming the garter around the body (T2)] and pigmentation stages [10 h after ecdysis (T3) and 24 h after ecdysis (T4)] to identify important genes and pathways underlying the development of green and brown pupa in the swallowtail butterfly *Papilio xuthus*. Combined comparisons of each developmental stage and each tissue under green and brown conditions, a total of 1042 differentially expressed genes (DEGs) and 430 different abundance proteins (DAPs) were identified. Weighted gene co-expression network analysis (WGCNA) and enrichment analysis indicate that these DEGs were mainly related to oxidation-reduction, structural constituent of cuticle, and pigment binding. Soft clustering by Mfuzz and enrichment analysis indicate that these DAPs are mainly involved in tyrosine metabolism, insect hormone biosynthesis, and melanogenesis. By homologous alignment, we further identified those genes encoding neuropeptides (51), GPCRs (116), G-proteins (8), cuticular proteins (226), chitinases (16), and chitin deacetylases (8) in the whole genome of *P. xuthus* and analyzed their expression profiles*.* Although we identified no gene satisfying with hypothesized expression profile of PCMH, we found some genes in the neuropeptide cascade showed differentially expressed under two pupal color conditions. We also found that Toll signaling pathway genes, juvenile hormone (JH) related genes, and multiple cuticular proteins play important roles in the formation of selective pupal colors during the prepupal-pupal transition. Our data also suggest that both green and brown pupa include complex pigment system that is regulated by genes involved in black, blue, and yellow pigments. Our results provide important insights into the evolution of pupal protective colors among swallowtail butterflies.

## Introduction

Phenotypic plasticity can be broadly defined as the ability of one genotype to produce more than one phenotype when exposed to different environments, as the modification of developmental events by the environment, or as the ability of an individual organism to alter its phenotype in response to changes in environmental conditions, and thus is a powerful means of adaptation (Kelly et al., 2012). Phenotypic plasticity in general and polyphenic development in particular are thought to play important roles in organismal diversification and evolutionary innovation (Moczek and Nijhout, 2002; Moczek, 2010). Color polyphenism, one famous example of phenotypic plasticity, is a common phenomenon at different developmental stages among insect taxa, and used as an important strategy to enable the avoidance of predation by predators (Hazel, 2002; Tanaka, 2004; Noor et al., 2008; Johansson and Nilsson-Ortman, 2013). Among them, the pupa of some butterflies such as Papilionidae, Pieridae, and Nymphalidae are reported to show color polyphenism (Wiklund, 1972; Smith, 1978; Smith, 1980; Maisch and Buckmann, 1987; Hazel, 1995, Hazel, 2002; Tanaka, 2004; Ferreira et al., 2006; Jones et al., 2007; Noor et al., 2008; Yamamoto et al., 2011; Kelly et al., 2012; Johansson and Nilsson-Ortman, 2013).

Larvae pupating on green twigs of host plant will likely form green pupae, while those pupating on dead branches are of the brown type (Ohnishi, 1959). Many swallowtail butterflies (Papilionidae) are reported to determinate their pupae color (green or brown etc.) depending on different environmental stimuli, such as temperature, relative humidity, wavelength of light, photoperiod, and background during pupation (Hiraga, 2005; Hiraga, 2006; Yamamoto et al., 2011). Tactile stimuli of pupating background received at pre-pupae stages both before and after girdling is regarded as the most critical factor for inducing the formation of brown pupae, while light, living plant leaves or 100% humidity can suppress the formation of brown pupae (Hiraga, 2006). Biochemically, green and brown pupae show different pigment composition in their cuticle. Cuticle is mainly composed of chitin and many kinds of cuticular proteins (Liu et al., 2018). The pigment of green pupae was a mixture of blue (bile pigment) and yellow (carotenoids) chromoproteins (Ohnishi, 1959; Riley et al., 1984; Yoda et al., 2020), while melanin, canthaxanthine, papilioerythrin (oxidized lutein) were found in brown pupae (Ohnishi, 1959). Physiologically, ligatured abdomens experiment reveals that neuroendocrine mechanism involved in the pupal color dimorphism in *Papilio xuthus* (Awiti and Hidaka, 1982). The brain of the *P. xuthus* received and integrated carefully environmental factors from pupation site during the pre-pupa stage by tactile signals, and synthesized and released a kind of hormone called pupa cuticle melanizing hormone (PCMH). This hormone was secreted from brain-suboesophageal ganglion and prothoracic ganglion complexes (Br-SG-TG1) and induced the formation of brown pupae (Yamanaka et al., 1999). In the nymphalids and pierides, the pupal color is regulated by a pupal melanization reducing factor (PMRF) by inhibiting pupal melanization (Yamanaka et al., 2004). PMRF or PMRF-like molecules in the nymphalids and pierides are similar to PCMH in papilionoids, but they show opposite effects on pupal color between two category butterflies (Starnecker and Hazel, 1999). PMRFs were possibly one kind of water-soluble neuropeptides limitedly distributing in lepidopteran insect (Maisch and Buckmann, 1987; Starnecker, 1996) and can induce up-regulation of intracellular cyclic adenosine monophosphate (cAMP) by binding to G-protein coupled receptor (GPCR) in target neurosecretory cells (Jones et al., 2007). Neuropeptides, commonly signalled by GPCRs, play a central role in the physiological processes around whole life of insects, such as controlling of development, reproduction, behavior, feeding and others (Tanaka et al., 2014; Bauknecht and Jekely, 2015; Yeoh et al., 2017). For molecular basis of pupal coloration, the only report was on pigmentation genes involved in the pupal coloration of *P. polytes*, including melanin synthesis genes, tyrosine hydroxylase and laccase 2 responsible for the formation of brown pupae, and multiple bilin binding protein (BBP)-related genes and multiple juvenile hormone binding protein (JHBP)-related genes responsible for the formation of green pupae (Yoda et al., 2020). Especially, the knowledge of the expression profiles of PCMH/PMRF and related genes/metabolic pathways between green and brown pupae are very limited.

To understand the molecular mechanism underlying pupal color switch (green or brown pupae) in butterflies, we focus on whether PCMH/PMRF and their cascade pathways, and pupal cuticle composition are different under different pupation conditions by combining RNA-sequencing (RNA-seq) and proteome sequencing (iTRAQ-seq) using *P. xuthus* as an experimental model. We firstly obtained 100% green and brown pupae under laboratory conditions. RNA-seq analyses of Br-SG-TG1, mesothoracic and metathoracic ganglia and all abdominal ganglia complexes (TG23-AG), and epidermis, and iTRAQ analyses of whole ganglion were employed underlying four developmental stages to explore differentially expressed genes and proteins under conditions of producing green and brown pupae. Weighted gene co-expression network analysis (WGCNA) and soft clustering with Mfuzz were explored to analyze the co-expression of these differentially expressed genes and proteins, respectively. We further genome-wide identified the genes encoding neuropeptides, G-protein, and GPCRs, and investigated their expression profiles at both transcriptional level and protein level. We also explored pigmentation related genes, genes encoding cuticular proteins, and chitin metabolism related genes responsible for the pupal color switch of *P. xuthus*. This study provides important molecular clues for further interpretation the evolutionary process of pupal protective colors among butterflies.

## Methods

### Insect Rearing and Sample Collection

Pupae of the swallowtails butterfly, *P. xuthus*, were provided by butterfly insectariums in the suburbs of Ya’an, Sichuan, China. Pupae were reared under the conditions of 26°C, 75% relative humidity and 18 h/6 h light/darkness. Emerged adults were crossed *via* hand pairing. After mating, females were placed in net rooms with host plants (*Zanthoxylum piperitum*) for oviposition. Eggs were collected and reared under the conditions of 60% humidity, 16 h light-27°C/8 h dark-25°C cycle. The hatched larvae were also reared in the same condition as eggs.

The pupation apparatus were modified with reference to the method by Hiraga (Hiraga, 2006) to obtained the polymorphic pupae. In details, we attempted to acquire green and brown pupae by controlling such environment factors as light, humidity, temperature, pupating background. However, we couldn’t consistently obtain 100% pupa with expected color when only one environment factor mentioned above was changed. Thus, we combined two strategies to ensure the development of expected pupal color. Firstly, we established two extremely different growing conditions that combined multiple environmental factors, including the green condition (GN: a transparent and smooth plastic container with the fresh host plant under the conditions of 100% humidity and 24 h light) and the brown condition (BN: the cone-shaped container folded with waterproof abrasive paper (240 mesh: Hubei Yuli Abrasive Belts Group, China) at 24 h darkness). To obtain green pupae and brown pupae, final-instar larvae after gut purge were put into the GN container and the BN container, respectively. Secondly, we ensured about half of each batch larvae in the same condition for RNA-seq/iTRAQ-seq materials were reared to at least 10 h after ecdysis when pupa color was fixed. If there is un-expected pupal color, the whole batch is discarded.

**FIGURE 1:**
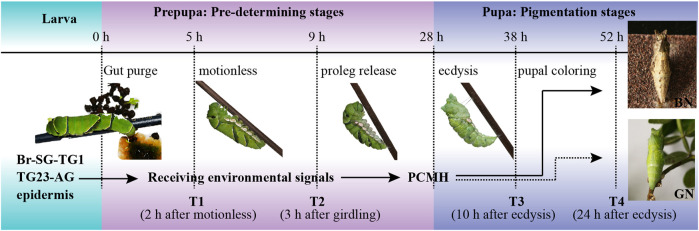
Developmental process of dimorphic pupal color formation in *Papilio xuthus*. The brain of pre-pupa receives and integrates carefully environmental factors from pupation site during the pre-determining stages by tactile signals, and synthesizes and releases a hormone called pupa cuticle melanizing hormone (PCMH) from brain-suboesophageal ganglion and prothoracic ganglion complexes (Br-SG-TG1). This hormone determines the color of the pupal body in pigmentation stages. BN, brown pupae; GN, green pupae; TG23-AG, the mesothoracic and metathoracic ganglia (TG23) and all abdominal ganglia (AG) complexes. The solid arrow indicates PCMH release, and the dotted arrow indicates no PCMH release. Samples in this study were collected from four time points, including T1 (pre-pupae, 2 h after gut purge) and T2 (proleg release, 3 h after forming the garter around the body) in pre-determining stages, T3 (pupal coloring, 10 h after ecdysis) and T4 (pupal coloration, 24 h after ecdysis) in pigmentation stages.

The samples for RNA-seq were collected at four time-points, including T1 (pre-pupae at 2 h after motionless), T2 (proleg release, 3 h after forming the garter around the body), T3 (pupal coloring, 10 h after ecdysis), and T4 (pupal coloration, 24 h after ecdysis) (Figure 1). For each individual, Br-SG-TG1 complex, TG23-AG complex, and whole epidermis were dissected under binocular stereomicroscope (Nikon, Japan). Non-targeted tissues around ganglion and impurity (e.g., trachea, fat and body fluids) around epidermis were removed by multiple washing using cold phosphate-buffered saline. The corresponding tissues from three individuals were mixed as one biological replicate, and three biological replicates were carried out for each sample. A total of 72 samples (two kind of pupal color, four time-points, three tissues, three biological replicates) were dissected for RNA-seq. For iTRAQ-seq, samples were collected from the same four time-points as in RNA-seq. i.e., T1, T2, T3, and T4 (Figure 1). The ganglions (Br-SG-TG1 and TG23-AG complexes) of 15 individuals were dissected and mixed together as one protein sample.

### Transcriptome Sequencing and Data Analysis

Total RNA was isolated using TRIzol reagent (Invitrogen, United States) according to the manufacturer’s instructions. The 350 bp insert size paired-end libraries were generated and sequenced using Illumina HiSeq4000 sequencers at Novogene (Tianjin, China). The quality of the pair-end raw reads with length of 150 bp was evaluated using FastQC (https://www.bioinformatics.babraham.ac.uk/projects/fastqc/). The adapter and low-quality reads (>10% Ns, or >30% low-quality with base quality <20) were filtered out using Trimmomatic v0.39 (Bolger et al., 2014). All cleaned reads were first mapped to the genome of *P. xuthus* (Li et al., 2015) using hisat2 (Kim et al., 2015) with the default parameters. The alignments were then passed to StringTie (Pertea et al., 2015; Pertea et al., 2016) to quantify the transcripts and estimate the abundances in each sample. The raw counts generated from StringTie were used as input for DESeq2. Differential expression analysis was performed using the DEGseq2 package (Love et al., 2014) based on the normalized raw counts for the tissue of interest at each time point. The criteria for screening significantly differentially expressed genes (DEGs) under green and brown pupa were |log2FoldChange| >1 and adjusted *P*-value < 0.05. Gene ontology (GO) and KEGG enrichment analysis of DEGs from each comparison were carried out using clusterProfiler package (Yu et al., 2012) in R software, respectively. *P*-value were corrected with Benjamini-Hochberg method and false discovery rate <0.05 was used as a threshold of significance. Cor function in R software with spearman correlation coefficient was used for correlation analysis.

Weighted gene co-expression correlation network analysis (WGCNA) was used to find modules of highly correlated genes in expression profile (Langfelder and Horvath, 2008). Each gene with total normalized counts (DEseq2) lower than 2 in our 72 samples was excluded. The appropriate soft power value was determined by a gradient method (the power value ranging from 1 to 20) and subsequently used for co-expression module construction. Modules were identified with a dynamic tree-cutting algorithm with a minimum module size of 30 genes and merged with MEDissThres parameter for 0.25. Module-trait associations were estimated using the correlation between the module eigengene and the phenotype. Gene ontology (GO) and KEGG enrichment analysis were carried out using clusterProfiler package (Yu et al., 2012) in R software and *P*-value were corrected with Benjamini-Hochberg and less than 0.05 was used as a threshold of significance.

### Proteome Sequencing and Data Analysis

#### Protein Preparation and iTRAQ Labeling

All processes of the protein preparation and iTRAQ labeling were carried out by the company of Genecreate (Wuhan, China) as described in our previous work (Zhang et al., 2020). Briefly, each biological sample was individually ground to powder in liquid nitrogen and incubated in Lysis buffer (7M Urea/2M Thiourea/4% SDS/40 mM Tris-HCl, pH 8.5/1 mM PMSF/2 mM EDTA) for 5 min, then 10 mM DTT (final concentration) were added to the sample. The suspension was sonicated for 15 min and then centrifuged at 4°C, 13,000 g for 20 min. The supernatant was mixed with 4 volumes of precooled acetone at −20°C overnight. After centrifugation, the protein pellets were air-dried and resuspended in 8 M urea/100 mM TEAB (pH 8.0). Protein samples were reduced with 10 mM DTT at 56°C for 30 min, alkylated with 50 mM iodoacetamide (IAM) for 30 min in the dark.100 μg of the proteins were extracted from each sample after grinded, incubated, sonicated, centrifuged, precooled, air-dried and resuspended, reduced and alkylated. Protein concentration was measured using Bradford method. Then, the purified proteins were used for tryptic digestion.

#### Mass Spectrometry Data Acquisition

The peptide samples were dissolved in 0.5 M TEAB and labelled using iTRAQ kits (Applied Biosystems, Foster City, CA). The mixed peptides were fractionated using the Ultimate 3000 HPLC system (Thermo DINOEX, United States). Mass spectrometry data were produced by the TripleTOF 5600 + liquid mass spectrometry system (SCIEX, United States) coupled with the Eksigent nanoLC system (SCIEX, United States). TripleTOF 5600plus liquid chromatography and mass spectrometry system (SCIEX) was used for mass spectrometry data acquisition.

#### Proteomics Data Processing

Protein identification and quantification were searched against the protein sets of *P. xuthus* (15,384 items) (Li et al., 2015) using ProteinPilot v4.5 software (AB Sciex 5600 plus), with the unused score ≥1.3 (which corresponds to proteins identified with ≥95% confidence). The correlation of quantitative results was evaluated using Pearson algorithm. The different abundance proteins (DAPs) were defined with a fold-change (FC) ≥1.5 or ≤0.67 and a *P*-value ≤ 0.05 (t-test of all comparison groups). The identified proteins were functional annotated based on the database of GO, COG and KEGG. DAPs were performed GO and KEGG pathway enrichment analysis (*P*-value < 0.05). The R package (https://www.r-project.org/) was used for statistical expression data and visualization. An R package termed Mfuzz implement soft clustering tools for the co-expression analysis of DAPs. (Kumar, 2007).

### Correlation Analysis for DEGs and DAPs

A comparative association analysis was conducted in the transcriptomic data from two ganglion complexes (Br-SG-TG1 and TG23-AG) and proteomic data from whole ganglion. For each gene that was expressed at transcriptional level, if its corresponding protein was expressed, it is regarded as correlation. For each gene that was expressed at protein level, if its corresponding transcript were expressed, it is also regarded as correlation. Furthermore, we screened DEGs and DAPs (cor-DEGs-DAPs) at each time-point. The function of cor-DEGs-DAPs were performed by BLASTP searching against the non-redundant (NR) protein database of NCBI with a cut-off E-value of 1e-5.

### Genome-wide Identification and Expression Analysis of Genes Possibly Involved in Pupal Color Switch

Homologous alignment was used to identify the candidate genes possibly involved in pupal color switch. Briefly, BLASTP searches (E-value < 1e-5) (Ye et al., 2006) were performed against the reference genome of *P. xuthus* using reported sequences of corresponding proteins from flybase (http://flybase.org/), NCBI (https://www.ncbi.nlm.nih.gov/), UniProt (https://www.uniprot.org/), and InsectBase 2.0 (http://v2.insect-genome.com/) as queries. Conserved domains were scanned by the Hidden Markov Model in HMMER v3.2.1 (Finn et al., 2015) with an E-value cutoff of 1e-5. After removing redundancy, candidate genes with the best hit score were retained and further confirmed using BLASTP program in the NCBI webserver (www.ncbi.nlm.nih.gov/blast).

Neuropeptides, GPCRs, and G-protein involve in the pupal color dimorphism by neuroendocrine mechanism (Awiti and Hidaka, 1982). The query sequences of neuropeptides were extracted from five arthropoda species, *Tribolium castaneum* (Li et al., 2008), *Drosophila melanogaster* (Hauser et al., 2006), *Bombyx mori* (Roller et al., 2008), *Apis mellifera* (Hummon et al., 2006), *Danaus plexippus* (Zhan et al., 2011). The signal peptide in the N-terminal of neuropeptides was predicted by the software SignalP v5.0 (http://www.cbs.dtu.dk/services/SignalP/) (Almagro Armenteros et al., 2019). The query sequences of GPCRs were extracted from *D. melanogaster* (http://flybase.org/) and *B. mori* (Fan et al., 2010). Protein structure information for all candidate GPCRs was predicted with TMHMM 2.0 (http://www.cbs.dtu.dk/services/TMHMM-2.0) and the genes with more than three transmembrane segments were remained. For G-proteins, six Gα genes from *D. melanogaster* (DmeGs: *CG2835*; DmeGi: *CG10060*; DmeGq: *CG17759*; DmeGo: *CG2204*; DmeGf: *CG12232*; Dmecta: *CG17678*) were used as queries. Those protein sequences with Gα domain (PF00503) were remained. Together with six *D. melanogaster* Gα genes, the candidate Gα were aligned using MAFFT (Katoh and Standley, 2013) with BLOSUM62 matrix and L-INS-i iterative refinement method, and the poorly aligned regions and gaps were removed using trimAl with settings “gt = 0.5.” The maximum likelihood (ML) tree was constructed using RAxML (Stamatakis, 2014) with settings “--auto-prot = ml -f a -x 12345 -N 100 -p 12345 -o *DmeGo* -m PROTGAMMAWAG”, and then visualized and modified using iTOL (Letunic and Bork, 2021).

Juvenile hormone (JH) and 20-hydroxyecdysone (20E) coordinate in the regulation of larval molting and metamorphosis in insects (Cheng et al., 2014; Li et al., 2021). The gene encoding 20E was identified in the part of neuropeptide identification. Genome-wide identification of genes involved in the biosynthesis, metabolism, and signaling of JH were referred to the method of Cheng et al. (2014).

Pigments, cuticular protein, and chitin are the main components of puparium (Futahashi et al., 2012; Liu et al., 2018; Yoda et al., 2020). So, we identified the genes related to these components. For pigmentation related genes, we investigated melanin-related genes that are involved in black pigmentation, including those encoding tyrosine hydroxylase, dopa decarboxylase, yellow, ebony, tan, and laccase 2 (Li et al., 2015; Yoda et al., 2020), bilin binding protein (BBP: blue pigment), yellow-related gene or carotenoid binding protein 1, and juvenile hormone binding protein (JHBP)-related genes (yellow pigment) that are involved in green pigmentation (Shirataki et al., 2010; Futahashi et al., 2012). In addition, the Toll signaling pathway genes that involved in red/black pigmentation (Nishikawa et al., 2013; KonDo et al., 2017) were also investigated. For cuticular proteins, CPR family genes with R&R Consensus (Rebers and Riddiford, 1988; Iconomidou et al., 1999) were further discriminated between the two subtypes at cuticleDB website (http://bioinformatics2.biol.uoa.gr/cuticleDB/index.jsp). The Tweedle motif was used to identify homologs of Tweedle genes (Guan et al., 2006). For the CPAP, CPF, and CPFL families, the conserved motif PF01607 and PF16042 were used to identify each family members, respectively. For chitinases, and chitin deacetylases, the conserved domains, including consensus catalytic domains of chitinases (GH18, [pfam00704]) and deacetylases (CE4-1, [cd10974] and CE4-2, [cd10975]) were used as queries to identify homologous genes in the genome of *P. xuthus*.

The core clock genes regulate circadian rhythm in *Drosophila* (Cyran et al., 2003; He et al., 2017). Six core clock genes found in the *D. melanogaster* were used as query to BLASTP (E-value <1e-5) search against the genome of *P. xuthus*.

All putative genes identified above were further screened their expression profiles between green and brown conditions in each tissue and each time-point at transcriptional and protein levels.

## Results

### Overview of the Transcriptomic Analysis

**FIGURE 2:**
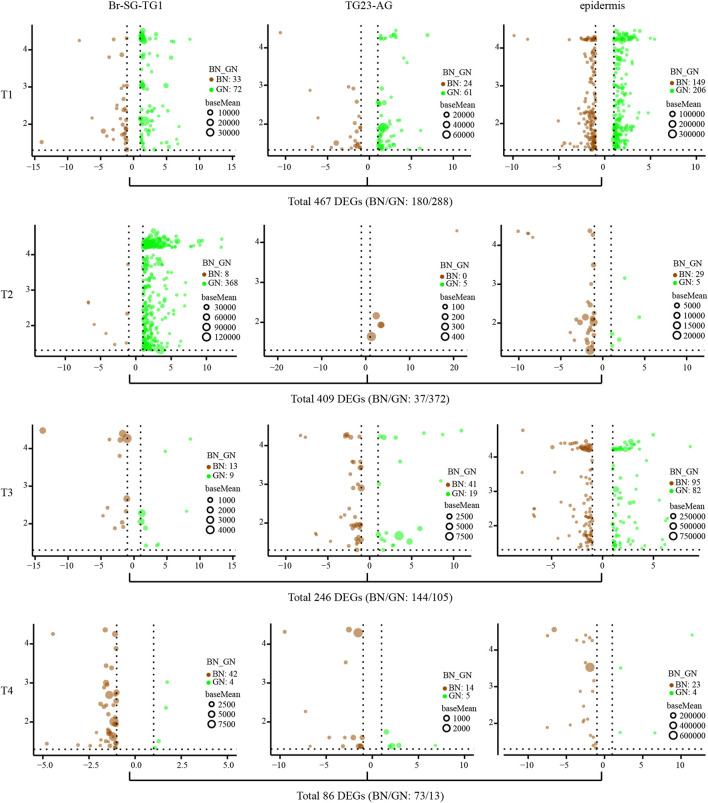
The analysis of differential expression genes (DEGs) of three kind of tissues (Br-SG-TG1, TG23-AG, and epidermis) under green and brown conditions at four investigated time-points (T1, T2, T3, and T4) in pupal color formation of *Papilio xuthus*. The x-axis and y-axis are log2 fold change and negative log10 transformed adjusted *P*-value (padj), respectively. Full information of tissues and periods refers to Figure 1.

The average number of clean bases per sample is 6.13 G, and their average mapping rate against the reference genome of *P. xuthus* is 83% (Supplementary Table S1). Correlation analysis of gene expression profiles indicates high consistency among different samples including ganglion (-1: Br-SG-TG1 and -2: TG23-AG) and epidermis (-3) (Supplementary Figure S1). The differently expressed genes (DEGs) between green and brown conditions vary in ganglion complexes (Br-SG-TG1 and TG23-AG) and epidermis during four developmental stages (T1–T4) (Figure 2 and Supplementary Table S2). A total of 467 (BN/GN: 180/288), 409 (BN/GN: 37/372), 246 (BN/GN: 144/105), and 86 (BN/GN: 73/13) DEGs were identified at T1, T2, T3, and T4, respectively. We performed GO and KEGG enrichment analysis of DEGs that obtained from each comparison (Supplementary Data S1). DEGs at T1 were involved to oxidation-reduction, iron ion binding, transporters, and juvenile hormone metabolism etc. DEGs at T2 were mainly associated with oxidation-reduction, iron ion binding, pigment binding, chitin metabolism related, extracellular region, fatty acyl-CoA reductase, tyrosinase etc. Enrichment of DEGs at T3 showed that oxidation-reduction, structural constituent of cuticle, pigment binding, and many enzymes were significantly enriched. DEGs at T4 were enriched in chitinase, glutathione S-transferase and transporter etc. All above suggested that these DEGs are likely to play different roles between green and brown conditions during prepupal-pupal transition.

**FIGURE 3:**
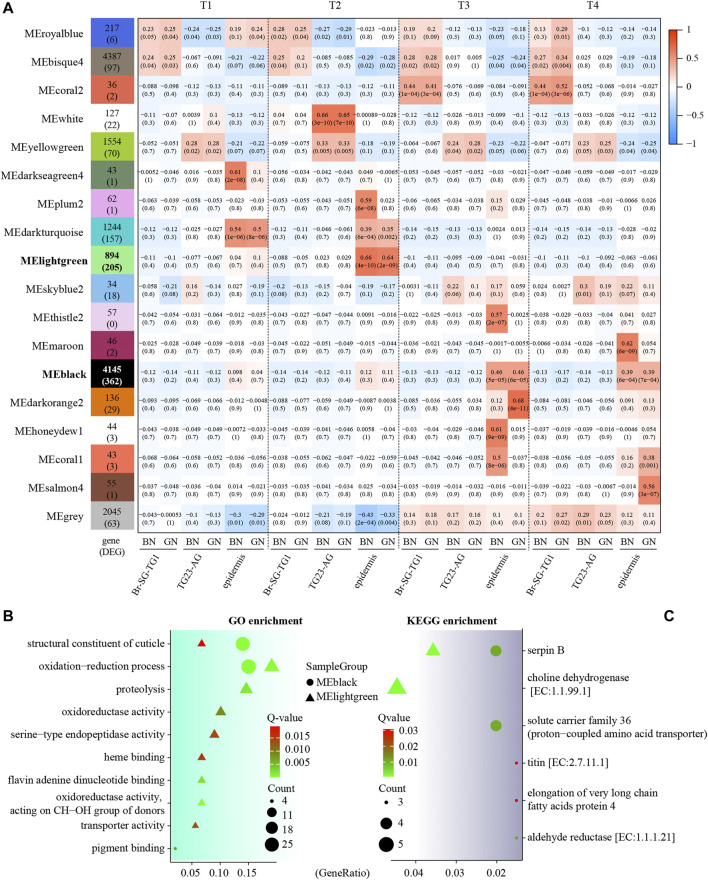
Module-trait associations of expression profiles in three kind of tissues (Br-SG-TG1, TG23-AG, and epidermis) in *Papilio xuthus* pupa color formation under green and brown conditions using weighted correlation network analysis (WGCNA). **(A)** Each cell contains the corresponding correlation with the *p*-value in parentheses. Full information of tissues and periods refers to Figure 1. The table is color-coded by the correlation according to the color legend. **(B,C)** GO and KEGG enrichment analysis of DEGs in MEblack and MElightgreen modules using clusterProfiler package. GeneRatio is the ratio of the number of genes enriched on GO or KEGG term to the number of genes input for enrichment analysis.

We further used WGCNA analysis to find modules of highly corrected genes in expression profile. Sample clustering with average method shows no obvious outliers (Supplementary Figure S2). After the optional threshold parameter β (*β* = 14, Supplementary Figure S3) was determined, the correlation coefficient among genes were transferred into the adjacent coefficient. The dissimilarity of the topological overlap matrix was further calculated, and 18 modules were finally gained (Supplementary Figure S2). We then correlated these modules to developmental stages and tissues under green and brown conditions (Figure 3A and Supplementary Data S2). We found that most of modules were significantly correlated (*P*-value < 0.05) to pupal development periods or tissues under two color conditions. For examples, MEdarkseagreen4 (in epidermis of BN) and MEdarkturquoise (in epidermis of both BN and GN) were highly correlated to early pre-pupa stage (T1). MEwhite (BN and GN in TG23-AG), MEplum2 (BN in epidermis), MEdarkturquoise (BN and GN in epidermis) was highly correlated to later pre-pupa stage (T2). Notably, we found that MElightgreen and MEblack included the most DEGs (362 and 205) between green and brown conditions. MElightgreen was highly correlated to epidermis of T2, and MEblack was highly correlated to epidermis of T3 and T4. GO enrichment analysis of these DEGs in MEblack and MElightgreen indicate that they are mainly related to oxidation-reduction, structural constituent of cuticle, pigment binding, etc. (Figure 3B and Supplementary Data S3). KEGG analysis showed that DEGs were associated with serpin B (Toll signaling pathway), choline dehydrogenase, solute carrier family 36, titin, elongation of very long chain fatty acids protein 4 and aldehyde reductase (Figure 3C and Supplementary Data S3). All these findings suggest that different pathways, such as oxidation-reduction, structural constituent of cuticle, Toll signaling pathway, and pigment binding, contributed to the formation of green and brown pupae in different development stages.

### Overview of the Proteomic Analysis and Combined Analysis of Transcriptomic and Proteomic Data

**FIGURE 4:**
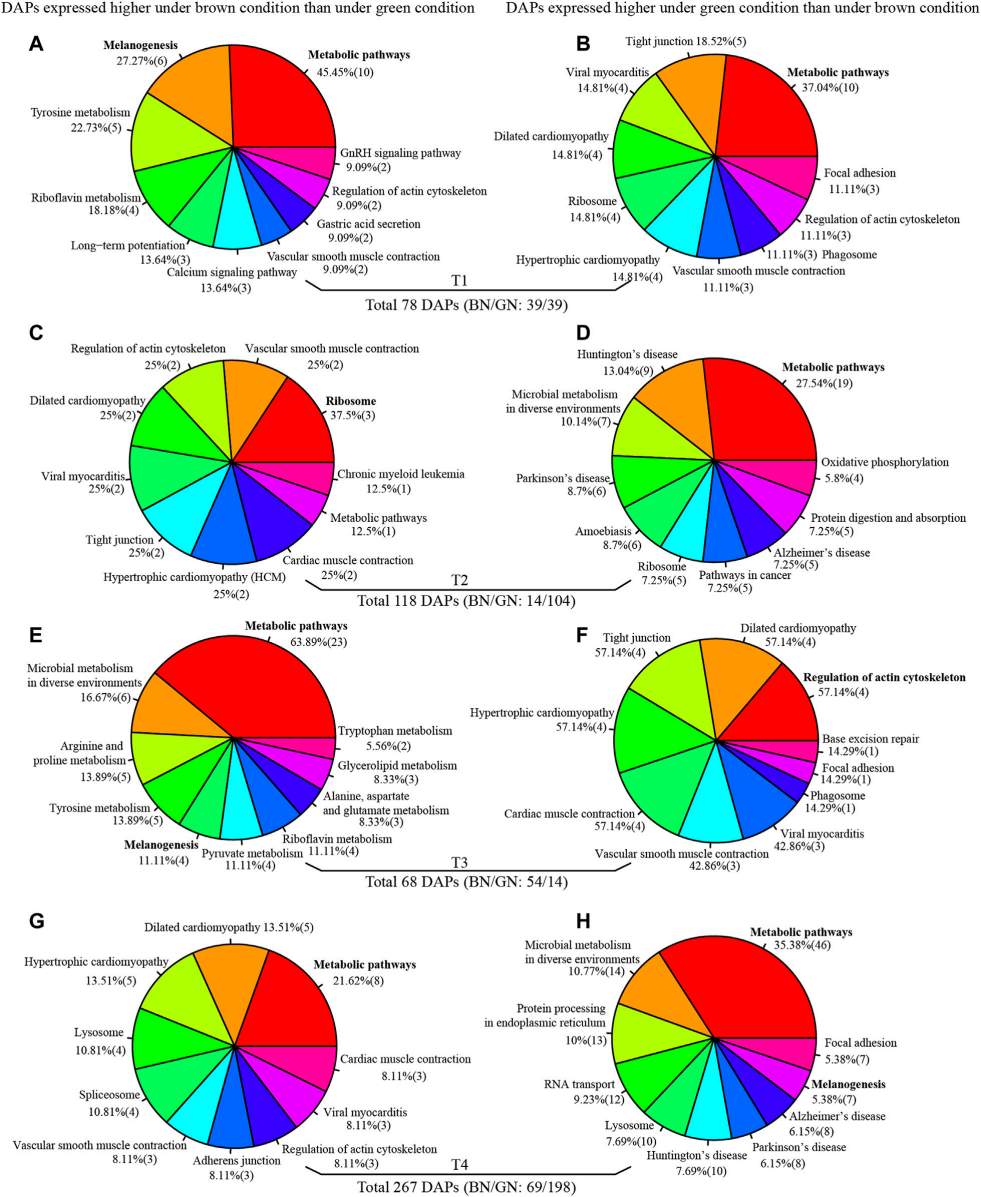
KEGG enrichment analysis of different abundance proteins (DAPs) at T1 **(A,B)**, T2 **(C,D)**, T3 **(E,F)**, and T4 **(G,H)**. Full information of periods refers to Figure 1.

To better understand the molecular mechanism of pupal color polyphenism from protein level, comparative proteomics was performed to identify the different abundance proteins (DAPs) under green and brown conditions using iTRAQ-seq. A total of 412,895 mass spectra were generated, and a total of 25,419 peptides and 3753 proteins were identified, in which 3056 identified proteins contained at least two unique peptides (Supplementary Table S3). The distribution of the unique peptides number, the peptide length, and the protein coverage are shown in Supplementary Figure S5. After quantification by ProteinPilot v4.5 software, 78, 118, 68, and 267 DAPs were identified at T1, T2, T3, and T4 under green and brown conditions (Figure 4 and Supplementary Data S4), respectively. GO enrichment analysis indicate that these DAPs identified from four developmental stages manly involve in metabolic process and cellular process (biological process), cell and cell part (cellular component), binding and catalytic activity (molecular function) (Supplementary Figure S6). KEGG enrichment analysis indicate that most DAPs at different time-points of both conditions involve in metabolic pathways (Figures 4A,B,D,E,G,H), except DAPs higher expression in brown condition than green condition at T2 (ribosome) (Figure 4C) and DAPs higher expression in green condition than brown condition at T3 (regulation of actin cytoskeleton) (Figure 4F). It is worth noting that many DAPs between green and brown conditions are associated with melanogenesis (Figures 4A,E,H), armadillo segment polarity protein, arylphorin, basic juvenile hormone-suppressible protein, calcyphosin-like protein, calmodulin, G-protein and phenoloxidase were up-regulated under the green condition (Supplementary Data S4). 1-phosphatidylinositol-4,5-bisphosphate phosphodiesterase, arylphorin, basic juvenile hormone-suppressible protein, calmodulin, and cAMP-dependent protein kinase were up-regulated under the brown condition (Supplementary Data S4), suggesting melanogenesis play roles in the formation of pupal color.

Mfuzz were used to cluster the expression profiles of DAPs at four developmental stages. Six clusters with completely inconsistent expression profiles were generated (Supplementary Figure S7A). Notably, we found the Toll signaling pathway related proteins (such as alpha-1-antitrypsin, antichymotrypsin, serpin, and sortilin-related receptor), cuticular proteins, bilin binding proteins (BBPs), and basic juvenile hormone-suppressible proteins displayed the similar expression profiles in cluster 1 (Supplementary Data S5). Enrichment analysis indicate that DAPs in cluster 1 involve in oxidation-reduction, proton transmembrane transport, tyrosinase, glutathione S-transferase, and Toll signaling pathway (serpin) (Supplementary Figure S7B).

In addition, we integrated the transcriptomic and proteomic data to investigate those genes differently expressed in both transcriptional and translational levels. Of the 15,174 genes expressed at transcriptional level, 148 genes were expressed at protein level. 3753 genes expressed at the protein level were also expressed at protein level. Notably, 10 genes were identified to differentially expressed at both transcriptional and protein levels (cor-DEGs-DAPs) (Supplementary Figure S8). Among them, four genes showed higher expression under the brown condition, including furry-like at T1 and another three (Cytochrome P450 9E2, dystonin, and fibrillin-1) at T4. On the other hand, four genes showed higher expression under the green condition, including defense protein at T1 and three genes (antichymotrypsin-2, alpha-1-antitrypsin, and two paramyosin) at T2 (Supplementary Figure S8).

### Expression Profiles of Genes Related to Neuropeptides, GPCRs and G-Proteins

To uncover the gene possibly encoding PCMH and the potential contribution of neuropeptides and their related protein to pupal color, genome-wide identification of neuropeptide or peptide hormones, and second messenger signaling cascades related to GPCRs and G-proteins were performed in *P. xuthus* genome. We identified 51 genes likely encoding neuropeptides or peptide hormones (Supplementary Data S6), 116 putative GPCRs genes (Supplementary Data S7) and 8 putative genes encoding G-protein (Supplementary Table S4) from *P. xuthus* genome. 34 neuropeptide genes contained a signal peptide that directed the protein to the secretory pathway. Unexpectedly, no up-regulated neuropeptide genes at transcriptional level or protein level was identified from ganglion (Br-SG-TG1 and TG2-3-A) under the brown condition, which is hypothesized as candidate genes encoding PCMH (Yamanaka et al., 1999). Interestingly, we identified three upregulated neuropeptide genes under the green condition. The genes encoding ecdysis triggering hormone (*Px_14591_unknow*) and bombyxin A-3-like (*Px_05886_unknow*) showed higher transcriptomic expression in epidermis under the green condition at T1 and T3, respectively (Supplementary Figures S9A,B). The gene encoding glycoprotein hormone alpha2 (*Px_16206_Gpa2*) was higher at T4 at protein level under the green condition (Supplementary Figure S9C). We also found some GPCRs showed differential expression under green and brown conditions at transcriptional level. The list under the green condition includes those genes encoding allatostatin-A receptor (Br-SG-TG1: T1), metabotropic glutamate receptor 3 (Br-SG-TG1: T2), G-protein coupled receptor Mth-like 3 (Br-SG-TG1: T2; epidermis: T3), and FMRFamide receptor (epidermis: T3) (Supplementary Figures S9D–G), and those under the brown condition include the genes encoding neuromedin-U receptor 2 and G-protein coupled receptor Mth2 both in epidermis of T1 (Supplementary Figures S10A,B). Besides, we identified two G-protein genes (the orthologs of *Drosophila Go* and *Gq*) showing higher proteomic expression under the green condition at T4 (Supplementary Figures S10C–E). These findings suggest that neuropeptide, GPCR and G protein cascades are involved in the pupal coloration before and after ecdysis.

### Expression Profiles of Genes involved in the Biosynthesis, Metabolism, and Signaling of Juvenile Hormone

**FIGURE 5:**
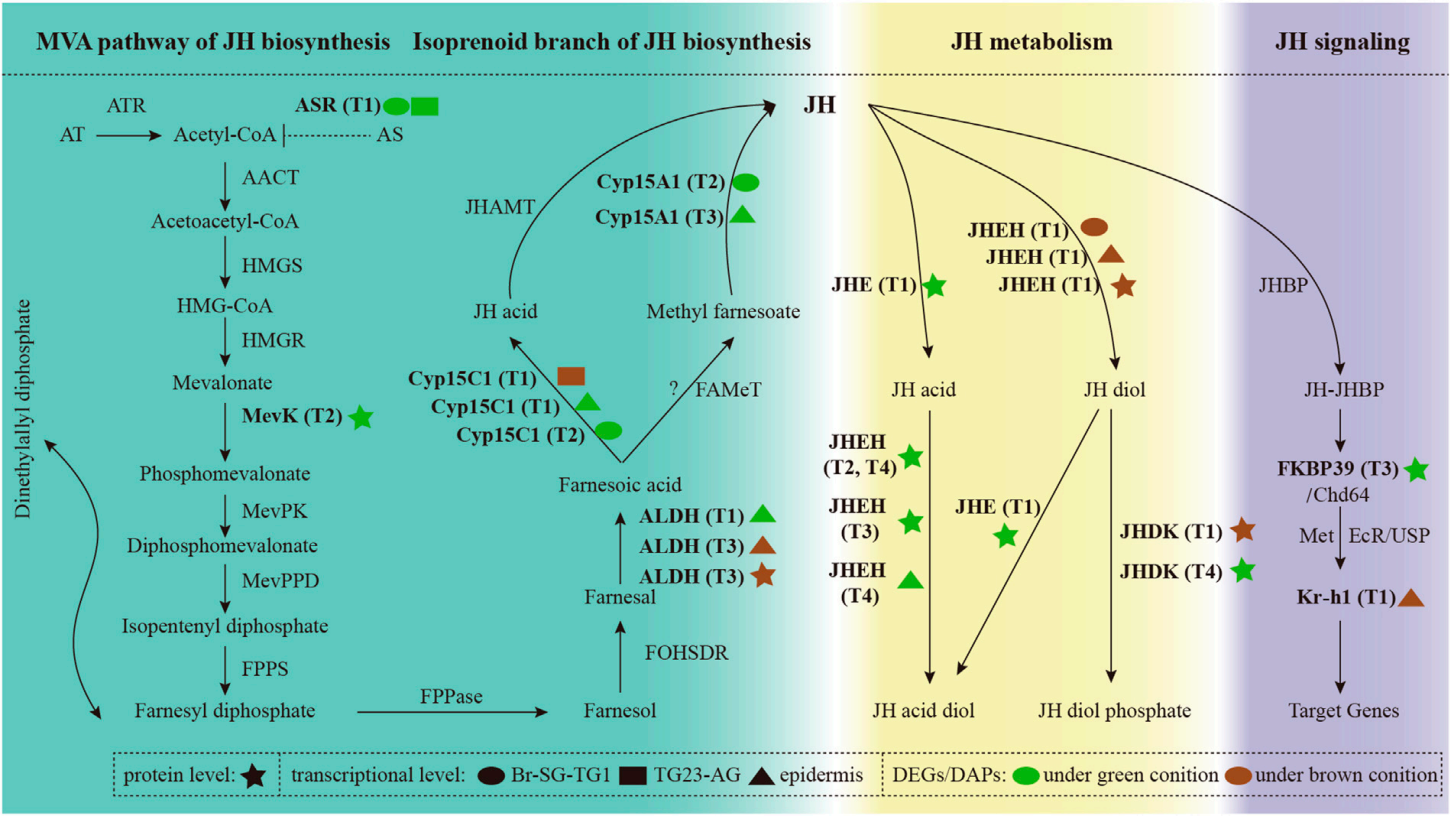
The expression profiles of genes involved in the biosynthesis, metabolism, and signaling of juvenile hormone. Full information of genes, tissues and periods refers to Supplementary Table S5 and Figure 1.

We identified 88 JH-related genes in the *P. xuthus* genome (Supplementary Table S5). Several genes that involved JH biosynthesis were differentially expressed, including three genes highly expressed under the green condition (allatostatin receptor, mevalonate kinase, and cytochrome P450 15A1), and two genes expressed under both condition (aldehyde dehydrogenase, and cytochrome P45015C1) (Figure 5), suggesting that the JH titer is different between green and brown conditions from larva to pupa.

Notably, genes involved in the JH metabolism were significantly differential expression in two pupal color conditions. Genes with higher expression under the green condition before ecdysis may be more inclined to decompose JH into JH acid diol. In contrast, genes with higher expression in brown condition before ecdysis are more likely to decompose JH into JH diol phosphate. JH signaling related genes were also differential expressed between green and brown conditions. In addition, 20E were not expressed significantly differential under green and brown conditions. Combined with the results mentioned in overview of the transcriptomic and proteomic analysis, these results indicate that JH related genes play important roles in metamorphic transition from larva to pupa.

### Expression Profiles of Genes Involved in Pigmentation

To determine the pigment related genes that involved in the pupal color formation, we analyzed their expression profiles under the green and brown pupal conditions, including black (melanin), blue, yellow, and black/red pigment-related genes (Supplementary Datas S8–S10; Supplementary Figures S11, S12).

At transcriptional level, we found that two melanin related genes were expressed higher under the brown condition at T1 (epidermis: *Px_06113_yellow−c*) and T4 (Br-SG-TG1: *Px_11801_yellow-e*) (Supplementary Figure S11A), and four melanin related genes were expressed higher under the green condition at T1 (TG23-AG: *Px_12819_yellow-c* and epidermis: *Px_15086_Ddc*), T2 (Br-SG-TG1: *Px_12819_yellow-c*, *Px_08231_ple*, *Px_15086_Ddc*, and *Px_17695_CG42345*), and T4 (TG23-AG: *Px_08231_ple*). Three tandem JHBPs that possibly involved in yellow pigmentation were expressed inconsistently under two pupal color conditions. Two JHBPs were expressed more highly in epidermis at T1 (*Px_13749_CG2650*), T3 and T4 (*Px_13748_CG10264*) under the brown condition, while JHBP (*Px_13747_CG10407*) exhibited the opposite pattern at T3 in epidermis under the green condition. For blue pigmentation, we found that multiple BBPs exhibited similar or opposite patterns under two pupal color conditions during whole pupation process, except from T2 under the brown condition and T4 under the green condition (Supplementary Figure S11). In addition, Two BBPs (*Px_15683_Nlaz* and *Px_15677_NLaz*) were also differentially expressed under green and brown conditions (Supplementary Figures S11B,C). The pupal pigmentation should appear to occur mainly after ecdysis (T3 and T4) because of new cuticle appeared transparent (Yoda et al., 2020). Regardless of the time-points before ecdysis (T1 and T2), the genes related to melanin, yellow and blue pigments are also differential expressed between green and brown conditions, suggesting that the co-expression of these genes is regulated to effectively produce pre-determined pupal color.

Toll signaling pathway genes were reported to participate in red/black pigmentation on *P. polytes* wings (Nishikawa et al., 2013). RNA-seq analysis found that 33 Toll signaling genes were differentially expressed under green and brown conditions (Supplementary Figure S12A; Supplementary Data S10). Among them, 21 genes were expressed more highly at T2 under the green condition in Br-SG-TG1, and six genes with higher expression under the brown condition were at T1 and T3. iTRAQ-seq analysis found that the expression of six Toll signaling genes induced at T2 and two genes induced at T3 were more highly under the green condition, while E3 ubiquitin-protein ligase herc4 (*Px_01959_CG9153*) and digestive cysteine proteinase (*Px_01499_26–29-p*) show higher expression under the brown condition (Supplementary Figure S12B; Supplementary Data S10). Combined with the results mentioned in overview of the transcriptomic and proteomic analysis, these results suggest that Toll signaling pathway genes play a synergistic or antagonistic role in the formation of pupal color.

### Expression Profiles of Genes Involved in Cuticle Formation

**FIGURE 6:**
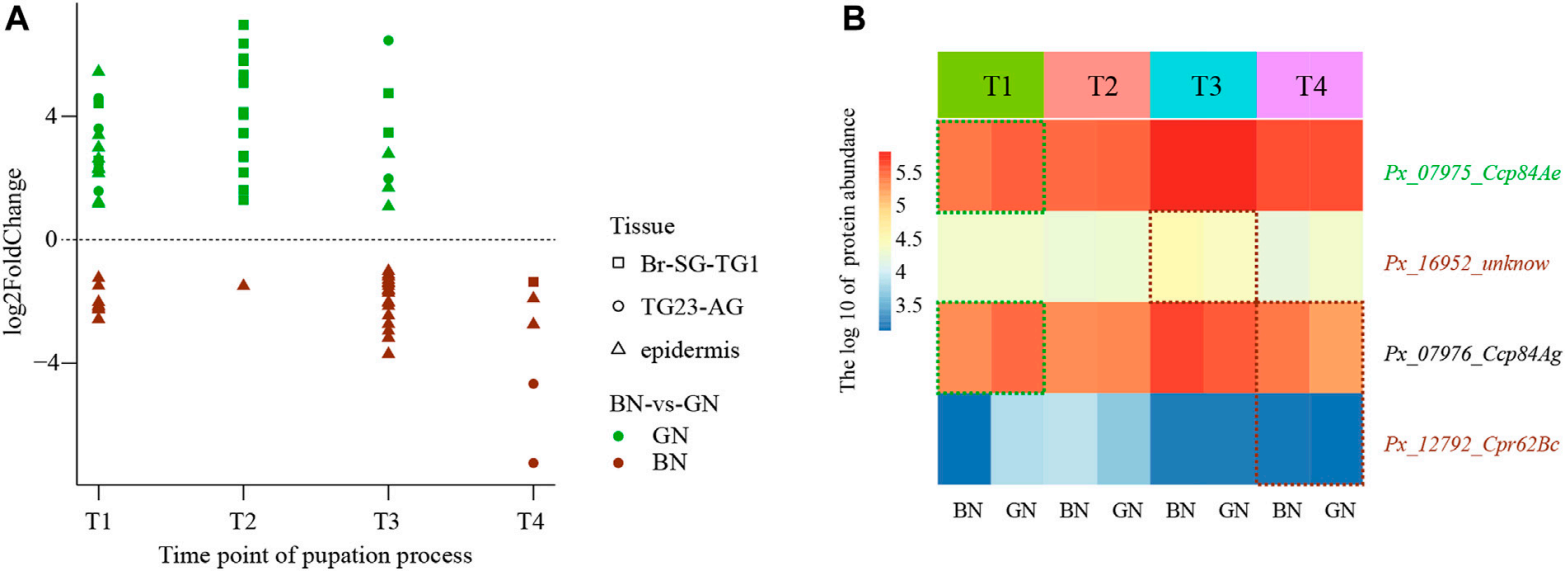
The expression profiles of cuticular proteins under green and brown conditions at transcriptional level or protein level. **(A)** The scatter plot of differential expression genes (DEGs) at transcriptional level. **(B)** Heatmap of the different abundance proteins (DAPs). Full information of tissues and periods refers to Figure 1.

To further trace the dynamic changes during the process of cuticle formation under green and brown conditions, we identified genes that encode cuticular protein, chitinase and chitin deacetylase in the genome of *P. xuthus* and analyzed their expression profiles. Totally 226 cuticular protein encoding genes, 16 chitinase encoding genes, and eight deacetylase encoding genes were identified (Supplementary Datas S11–S13). Among these, 60 cuticular proteins (BN/GN: 36/38) were differentially expressed in different stages and tissues at transcriptional level (Figure 6A and Supplementary Data S12). Cuticular proteins that expressed higher under the green condition were dispersed in the three tissues of T1 and T3. 16 cuticular proteins were expressed more highly at T2 of Br-SG-TG1 under the condition of green pupa, while the expression of many homologs was higher in the epidermis at T1, T2, and T3 under the brown condition. Four cuticular proteins were also differentially expressed at protein level (Figure 6B). In addition, six chitinases were differentially expressed at transcriptional level and one chitinase were differentially expressed at protein level (Supplementary Figure S13). Except chitinase (*Px_14677_Cht2*) that was expressed more highly at T4 in TG23-AG under the brown condition than green condition (Supplementary FigureS13B), other genes were not differentially expressed after ecdysis between green and brown conditions (Supplementary Figures S13B–F). One chitin deacetylase (*Px_15224_verm*) were differentially expressed before ecdysis (Supplementary Figure S14G). Combined with the results mentioned in overview of the transcriptomic and proteomic analysis, these results indicate that cuticular proteins contribute to the difference of cuticle composition between green and brown pupa.

## Discussion

Pupal color polyphenism of buttefly is an adaptive and ecologically important trait that is often involved in prey-predator interactions. In this study, we stably obtained green and brown pupae by simulating a similar pupation apparatus as Hiraga (2006) and combinating several environment factors, which ensures us to perform of RNA-seq and iTRAQ-seq at four important developmental stages, including the pre-determining stages (T1 and T2) and the pigmentation stages (T3 and T4) (Figure 1). The differences in light environment may affect the developmental trajectory on gene expression. Since light cycle entrains the circadian clock, and gene expression is circadian (Purushothaman et al., 2015; He et al., 2017; Krzeptowski et al., 2018; Niepoth et al., 2018). We investigated the expression of six transcription factors: Period (*Per*), Timeless (*Tim*), Cycle (*Cyc*), Clock (*Clk*), Vrille (*Vri*), and Par Domain Protein 1 (*Pdp1*) that primarily regulated the circadian rhythm (Supplementary Data S14) (Cyran et al., 2003). No genes or proteins were differentially expressed under the green condition verse under the brown condition. Therefore, we can basically rule out the influence of light environment on our experiment.

In the pre-determining stages (T1 and T2), larve would release the accumulated neuropeptide PCMH from Br-SG-TG1 to induce the formation of brown pupa (Yamanaka et al., 1999). Neuropeptides act on target cells by interacting with the complex of GPCRs and G-proteins, leading to the regulation of second messenger signaling cascades. Neuropeptides can exert a variety of effects (Wu et al., 2015; Schoofs et al., 2017) by this cascades. In this study, we identified 51 genes which likely encode neuropeptides or peptide hormones in *P. xuthus* (Supplementary Data S6). Only three neuropeptides (bombyxin A-3-like, ecdysis triggering hormone, and glycoprotein hormone alpha2) were expressed higher under the green condition than under the brown condition (Supplementary Figure S9). We did not find the corresponding gene encoding PCMH based on the expression profiles at transcriptional or protein level. With the development of sequencing technology, the power of single cell RNA sequencing combined with spatial transcriptomics can provide comprehensively insights for this neuroendocrine system that switch of pupal color polyphenism. Identifying PCMH itself by using interdisciplinary approaches may also help to uncover the switch of pupal color polymorphism. In addition, stage-specific changes in the titers of JH and 20E (two endocrine hormones) orchestrate molting, growth, metamorphosis in insects (Cheng et al., 2014). Changes in the expression of JH-related genes under green and brown conditions indicate that JH play important roles in the formation of two pupal color (Figure 5).

Pigmentation is located mainly in the new formated cuticle (Yoda et al., 2020). The expression of melanin-related genes, BBPs and JHBP or carotenoid binding protein 1 involved in black, blue, and yellow pigmentation, respectively (Shirataki et al., 2010; Yoda et al., 2020). In the pigmentation stages (T3 and T4), our results shown that tyrosine hydroxylase (*Px_08231_ple*) in melanin pathways was involved in the green pigmentation at T4 (Supplementary Figure S11A). Combining with the pathway for cuticular pigmentation in insects (Barek et al., 2018), we suppose that dopamine may be converted to colorless cuticle to participate in or converted to eumelanin to deepen the formation of green pupae. This expression pattern coincide with the dark green regions in the fifth instar of *Papilio* species (Shirataki et al., 2010). For blue pigmentation, the expression of multiple BBPs were tissue-specific and period-specific. Yoda et al. (2020) found that five BBP genes (1, 2, 4, 5, and 6) were highly expressed in the green-conditioned pupae only at the pupation stage (24–30 h after gut purge) in *Papilio polytes*. Five orthologous BBPs (*Px_15674_unknow*, *Px_15677_Nlaz*, *Px_15678_Nlaz*, *Px_15679_Nlaz*, and *Px_15681_Nlaz*) were also highly expressed in green-conditioned pupae at T3 in *P. xuthus* (Supplementary Figure S11), suggesting a modular fashion regulated these coordinated genes. Notably, we found two BBPs (*Px_15679_NLaz* and *Px_15682_Nlaz*) exhibited the opposite pattern in the brown-conditioned pupa during the pigmentation stages (Supplementary Figure S11A), suggesting that blue coloration formed by redundant functions of BBPs may also be part of the pigment system of brown pupa. For yellow pigmentation, the expression of JHBP (*Px_13748_CG10264*) was induced in epidermis during the pigmentation stages more highly under the brown condition than under the green condition (Supplementary Figure S11A). Another JHBP (*Px_13747_CG10407*) exhibited the opposite pattern at T3 (Supplementary Figure S11A). In addition, Toll signaling pathway related genes that involved in red/black pigmentation (Nishikawa et al., 2013; KonDo et al., 2017) were also participate in the formation of pupal color. In summary, we speculate that green or brown pupa is a complex pigment system that is regulated by genes involved in black, blue, and yellow pigments.

Cuticular proteins and chitin bind with each other to keep stable of cuticle structure to maintain the insect physical function (Moussian, 2010). Considering the new cuticle should be formated after ecdysis (T3 and T4), we identified genes encoding cuticular protein, chitinases and deacetylases, and investigated their expression profiles in epidermis after ecdysis. Three cuticular proteins and 24 cuticular proteins were expressed more highly under the green condition and brown condition, respectively (Figure 6A). All identiflied 16 chitinases and 8 deacetylases expression were no significant differences in epidermis after ecdysis between green and brown conditions (Supplementary Figure S13). Thus, we speculate the main differences in cuticle composition were induced by the expression of cuticular proteins between green pupa and brown pupa.

## Conclusion

We integrated the results from RNA-seq and iTRAQ-seq to explore the molecular mechanism of pupal color switch in *P. xuthus* butterflies. Through comparing the expression profiles between green condition and brown condition at transcriptional and protein levels, a total of 467, 409, 246, and 86 differential expression genes DEGs were identified during T1, T2, T3, and T4 at transcriptional level, respectively. 78, 118, 68, and 267 DAPs were identified during T1, T2, T3, and T4 at protein level, respectively. The dynamic changes of those genes/proteins exhibited diverse patterns during different development stages and tissues. In the pigmentation stages, pupal color is mixed with a variety of pigments, such as black, yellow, and blue pigments. Toll signaling pathway were involved in the formation of pupae color. Cuticular proteins contribute to the difference of cuticle composition between green and brown pupa. In the pre-determining stages, Genes involved in neuropeptide cascade were differentially expressed under the two pupal color conditions. Abroad range of studies will be required to better understand the neuroendocrine mechanism under the pupal color dimorphism. These findings will promote further exploring the molecular basis underlying the evolutionary process of pupal protective colors among butterflies and contribute to butterfly diversity conservation.

## Data Availability

The datasets presented in this study can be found in online repositories. The names of the repository/repositories and accession number(s) can be found below: https://www.ncbi.nlm.nih.gov/, PRJNA767225 http://www.proteomexchange.org/, PXD028820.
