# Supplementary material for: Integrated Analysis of Transcriptome and Proteome to Reveal Pupal Color Switch in Papilio xuthus Butterflies
Source: Front Genet. 2022 Feb 3;12:795115. doi: 10.3389/fgene.2021.795115 (PMC8852814; doi:10.3389/fgene.2021.795115)
Supplement: Supplementary file 1 [file DataSheet2.pdf]

Supplementary Material

Table S1. Statistics of the quality and the mapping rates of RNA-seq reads for *Papilio xuthus* (Px).

Sample	Library	Raw Reads	Clean Reads	Filter Reads	Raw Base (G)	Clean Base (G)	Effective Rate (%)	Error Rate (%)	Q20 (%)	Q30 (%)	GC Content (%)	Mapping Rate against Genome (%)
Px-BN-T1-A-1	RRA60067-S	15946530	15674666	13987423	4.78	4.70	98.30	0.03	96.57	91.59	46.12	85.18
Px-BN-T1-B-1	RRA60068-S	17715795	17534914	15743873	5.32	5.26	98.98	0.03	96.28	91.05	45.70	84.80
Px-BN-T1-C-1	RRA60069-S	20282300	20131110	17960828	6.08	6.04	99.25	0.03	96.38	91.47	45.65	85.45
Px-BN-T2-A-1	RRA60070-S	20010951	19879943	17794657	6.00	5.96	99.35	0.03	96.03	90.88	42.93	84.16
Px-BN-T2-B-1	RRA60071-S	21459705	21300327	18846394	6.44	6.39	99.26	0.03	96.39	91.44	45.91	85.47
Px-BN-T2-C-1	RRA60072-S	20297508	20076957	17786286	6.09	6.02	98.91	0.03	96.38	91.45	46.29	84.45
Px-BN-T3-A-1	RRA60073-S	21526680	21322587	18492351	6.46	6.40	99.05	0.03	96.99	92.66	46.20	85.00
Px-BN-T3-B-1	RRA60074-S	22041847	21697125	18732804	6.61	6.51	98.44	0.03	96.89	92.49	45.61	84.94
Px-BN-T3-C-1	RRA60075-S	22892104	22577595	19620210	6.87	6.77	98.63	0.03	97.12	92.91	46.20	84.66
Px-BN-T4-A-1	RRA60076-S	21108415	20952463	18284170	6.33	6.29	99.26	0.03	96.73	92.12	45.62	84.86
Px-BN-T4-B-1	RRA60077-S	25366967	25161861	21674509	7.61	7.55	99.19	0.03	96.98	92.63	46.04	85.06
Px-BN-T4-C-1	RRA60078-S	20294381	20084252	17619861	6.09	6.03	98.96	0.03	96.66	92.04	46.34	84.97
Px-BN-T1-A-2	RRA60079-S	25703838	25531129	21736305	7.71	7.66	99.33	0.03	96.84	92.36	46.27	84.77
Px-BN-T1-B-2	RRA60080-S	22943551	22578824	19935398	6.88	6.77	98.41	0.03	96.38	91.40	46.81	84.96
Px-BN-T1-C-2	RRA60081-S	16492835	16385438	14988470	4.95	4.92	99.35	0.04	93.97	85.44	46.42	84.43
Px-BN-T2-A-2	RRA60082-S	20337103	20008934	18249544	6.10	6.00	98.39	0.04	93.91	85.41	45.22	83.37
Px-BN-T2-B-2	RRA60083-S	20976695	20675977	18589787	6.29	6.20	98.57	0.04	94.58	86.46	46.59	84.60
Px-BN-T2-C-2	RRA60084-S	19809100	19664029	18604899	5.94	5.90	99.27	0.04	93.86	85.33	45.52	86.59
Px-BN-T3-A-2	RRA60085-S	22427053	22234296	21039705	6.73	6.67	99.14	0.04	93.84	85.25	45.69	86.44
Px-BN-T3-B-2	RRA60086-S	18658279	18449496	17421836	5.60	5.54	98.88	0.04	93.66	84.95	46.25	86.23
Px-BN-T3-C-2	RRA60087-S	20202624	20026223	18710338	6.06	6.01	99.13	0.05	93.55	84.40	46.91	87.14
Px-BN-T4-A-2	RRA60088-S	17320278	17119062	15687640	5.20	5.14	98.84	0.04	94.22	85.69	46.44	84.35

Px-BN-T4-B-2	RRA60089-S	23236962	23078742	20726637	6.97	6.92	99.32	0.04	94.61	86.56	45.69	84.55
Px-BN-T4-C-2	RRA60090-S	18365478	18262554	16308339	5.51	5.48	99.44	0.04	94.99	87.23	42.33	84.88
Px-BN-T1-A-3	RRA60101-S	22669676	22473885	20234727	6.80	6.74	99.14	0.04	94.09	85.53	46.50	82.09
Px-BN-T1-B-3	RRA60102-S	21676911	21481501	19929227	6.50	6.44	99.10	0.05	93.46	84.73	48.62	84.72
Px-BN-T1-C-3	RRA60103-S	17793059	17596095	15910013	5.34	5.28	98.89	0.04	94.13	85.80	48.80	82.79
Px-BN-T2-A-3	RRA60104-S	22854649	22646541	21117719	6.86	6.79	99.09	0.05	93.47	84.48	47.95	82.95
Px-BN-T2-B-3	RRA60105-S	22717957	22399171	20827357	6.82	6.72	98.60	0.05	93.48	84.80	49.27	84.36
Px-BN-T2-C-3	RRA60106-S	25327634	25040171	23614349	7.60	7.51	98.87	0.04	94.08	85.72	48.69	82.72
Px-BN-T3-A-3	RRA60107-S	18416742	18082330	14901593	5.53	5.42	98.18	0.04	94.28	87.72	57.81	68.89
Px-BN-T3-B-3	RRA60108-S	24770658	24370211	22370206	7.43	7.31	98.38	0.07	91.38	81.47	57.64	79.58
Px-BN-T3-C-3	RRA60109-S	21487991	21151580	19315948	6.45	6.34	98.43	0.07	90.78	80.37	58.05	79.11
Px-BN-T4-A-3	RRA60110-S	18257230	14940393	12771458	5.48	4.48	81.83	0.06	93.76	83.97	55.62	79.83
Px-BN-T4-B-3	RRA60111-S	24915840	24543727	19562037	7.47	7.36	98.51	0.04	94.25	87.70	56.95	72.44
Px-BN-T4-C-3	RRA60112-S	29254752	28936149	27127973	8.78	8.68	98.91	0.06	93.06	84.24	55.97	83.07
Px-GN-T1-A-1	RRA59761-S	21735720	20200512	18006853	6.52	6.06	92.94	0.03	95.50	89.41	45.62	84.96
Px-GN-T1-B-1	RRA59762-S	27694580	27408610	24589056	8.31	8.22	98.97	0.04	93.95	86.78	45.34	82.91
Px-GN-T1-C-1	RRA59763-S	31100160	15382545	13576004	9.33	4.62	49.46	0.03	95.66	89.91	47.70	84.55
Px-GN-T2-A-1	RRA59764-S	23456959	21791218	19516552	7.04	6.54	92.90	0.04	95.17	88.78	45.50	84.60
Px-GN-T2-B-1	RRA59765-S	19155614	17803853	16085025	5.75	5.34	92.94	0.04	95.14	88.77	44.97	84.81
Px-GN-T2-C-1	RRA59766-S	21566227	20046868	18072313	6.47	6.01	92.95	0.03	95.25	88.91	45.28	84.59
Px-GN-T3-A-1	RRA59767-S	19490089	18100757	16332052	5.85	5.43	92.87	0.03	95.53	89.43	45.30	84.76
Px-GN-T3-B-1	RRA59768-S	28331943	13996324	12469986	8.50	4.20	49.40	0.03	95.69	89.97	46.39	84.61
Px-GN-T3-C-1	RRA59769-S	25306282	25054455	21995896	7.59	7.52	99.00	0.03	95.59	89.78	45.78	83.68
Px-GN-T4-A-1	RRA59770-S	21556170	21318726	18941788	6.47	6.40	98.90	0.03	95.33	89.23	45.48	83.57
Px-GN-T4-B-1	RRA59771-S	23100591	22836796	20035884	6.93	6.85	98.86	0.03	95.57	89.72	45.02	83.95
Px-GN-T4-C-1	RRA60054-S	22932563	22761027	20102462	6.88	6.83	99.25	0.03	95.81	90.41	44.55	84.15
Px-GN-T1-A-2	RRA60055-S	17300169	17184540	15333163	5.19	5.16	99.33	0.03	96.00	90.65	45.77	84.75
Px-GN-T1-B-2	RRA60056-S	18451425	18281035	16226082	5.54	5.48	99.08	0.03	96.16	90.81	45.55	85.08
Px-GN-T1-C-2	RRA60057-S	15385088	15193836	13572515	4.62	4.56	98.76	0.03	96.25	90.95	45.84	84.85
Px-GN-T2-A-2	RRA60058-S	20772170	20504545	18209250	6.23	6.15	98.71	0.03	95.90	90.33	45.97	83.77
Px-GN-T2-B-2	RRA60059-S	16544635	16232023	14346055	4.96	4.87	98.11	0.03	96.15	90.77	47.27	84.82
Px-GN-T2-C-2	RRA60060-S	18270718	17981455	16070395	5.48	5.39	98.42	0.03	96.20	90.82	45.89	84.61

Px-GN-T3-A-2	RRA60061-S	17771648	17615203	15716311	5.33	5.29	99.12	0.03	96.19	90.88	45.88	83.55
Px-GN-T3-B-2	RRA60062-S	19030908	18858889	17805831	5.71	5.66	99.10	0.03	96.05	90.61	45.38	86.30
Px-GN-T3-C-2	RRA60063-S	21022683	20841725	18516121	6.31	6.25	99.14	0.03	96.12	90.96	45.97	83.80
Px-GN-T4-A-2	RRA60064-S	17903120	17647601	15586270	5.37	5.29	98.57	0.03	96.22	91.11	46.71	83.63
Px-GN-T4-B-2	RRA60065-S	21920933	21641087	18931018	6.58	6.49	98.72	0.03	96.44	91.56	45.93	83.98
Px-GN-T4-C-2	RRA60066-S	16505493	16215228	14360901	4.95	4.87	98.24	0.03	96.55	91.78	45.80	83.79
Px-GN-T1-A-3	RRA60091-S	20521417	20179041	18321920	6.16	6.05	98.33	0.04	94.28	86.02	47.06	82.04
Px-GN-T1-B-3	RRA60092-S	20006668	19689780	17905250	6.00	5.91	98.42	0.04	93.57	84.78	47.40	82.68
Px-GN-T1-C-3	RRA60093-S	18407835	18202574	16723876	5.52	5.46	98.88	0.06	92.80	83.38	47.11	79.75
Px-GN-T2-A-3	RRA60094-S	20231175	20012142	18347441	6.07	6.00	98.92	0.05	93.19	83.96	48.12	81.40
Px-GN-T2-B-3	RRA60095-S	16837850	16445113	14869730	5.05	4.93	97.67	0.04	94.60	86.84	48.00	82.62
Px-GN-T2-C-3	RRA60096-S	21478190	21198030	19406159	6.44	6.36	98.70	0.04	93.74	84.84	47.48	79.56
Px-GN-T3-A-3	RRA60097-S	24462686	24096674	20391345	7.34	7.23	98.50	0.06	92.07	82.44	55.02	75.71
Px-GN-T3-B-3	RRA60098-S	23458552	23087488	19022631	7.04	6.93	98.42	0.06	92.84	83.73	54.70	77.04
Px-GN-T3-C-3	RRA60099-S	25093568	24708801	21226073	7.53	7.41	98.47	0.07	91.75	82.02	56.17	72.71
Px-GN-T4-A-3	RRA60100-S	23681791	23313918	21266509	7.11	6.99	98.45	0.07	91.34	81.31	56.38	79.91
Px-GN-T4-B-3	RRA60113-S	25635170	25320554	20581216	7.69	7.60	98.77	0.06	92.02	82.58	56.70	73.69
Px-GN-T4-C-3	RRA60114-S	20471004	16465848	14178044	6.14	4.94	80.43	0.06	93.86	84.19	54.54	80.74
**Average**	**NA**	**21279887**	**20440126**	**18234206**	**6.38**	**6.13**	**96.55**	**0.04**	**94.84**	**87.89**	**47.89**	**83.00**

Raw reads: the number of raw reads sequenced. Clean reads: the number of clean reads obtained after filtering. Filter reads: the number of further filter clean reads using Trimmomatic v0.39. Raw bases: total base of sequencing raw data. Clean bases: total base of filtered data. Q20 and Q30: the percentage of bases with phred value greater than 20 and 30 in the total base. GC: percentage of G/C base number in total base number. BN: brown pupae. GN: green pupae. T1: pre-pupae at 2 h after gup purge. T2: proleg release (3 h after forming the garter around the body). T3: pupal coloring (10 h after ecdysis). T4: pupal coloration (24 h after ecdysis). A, B and C: biological replicates. -1: brain-suboesophageal ganglion and prothoracic ganglion complexes (Br-SG-TG1); -2: the mesothoracic and metathoracic ganglia (TG23) and all abdominal ganglia (AG) complexes (TG23-AG); -3: epidermis.

Table S2. Statistics of the number differentially expressed genes (DEGs) between green (GN) and brown (BN) conditions of RNA-seq.

Period	Tissue	Comparison	Up-regulation in BN	Up-regulation in GN	Total DEGs in each comparison	Total DEGs in each period (BN: GN)
T1	Br-SG-TG1	BN vs GN	33	72	105	
T1	TG23-AG	BN vs GN	24	61	85	467 (180: 288)
T1	epidermis	BN vs GN	149	206	355	
T2	Br-SG-TG1	BN vs GN	8	368	376	
T2	TG23-AG	BN vs GN	0	5	5	409 (37: 372)
T2	epidermis	BN vs GN	29	5	34	
T3	Br-SG-TG1	BN vs GN	13	9	22	
T3	TG23-AG	BN vs GN	41	19	60	246 (144: 105)
T3	epidermis	BN vs GN	95	82	177	
T4	Br-SG-TG1	BN vs GN	42	4	46	
T4	TG23-AG	BN vs GN	14	5	19	86 (73: 13)
T4	epidermis	BN vs GN	23	4	27	

BN: brown pupae. GN: green pupae. T1: pre-pupae at 2 h after gup purge. T2: proleg release (3 h after forming the garter around the body). T3: pupal coloring (10 h after ecdysis). T4: pupal coloration (24 h after ecdysis).

Table S3. Statistics of protein identification information.

Sample	Total spectra	Identification of spectra *	Identification of peptide *	Identification of protein *	Unique-2 peptides**
ALL	412895	125672	25419	3753	3056

* indicates that the reliability is at least 95%, and ** indicates the number of identified proteins containing at least 2 unique peptides.

Table S4. Homology comparison results of G-protein between *Drosophila melanogaster* (fly) and *Papilio xuthus* (Px).

Fly ID	Gene ID	Identify	Match	Mismatch	Gap openings	Fly start	Fly end	Px start	Px end	E-value	Bit score
cta-PA_FBgn0000384	*Px_05030_cta*	67.38	374	116	5	86	457	6	375	1E-171	485
Galphaf-PA_FBgn0010223	*Px_14775_Galpha73B*	49.31	361	171	2	45	399	32	386	7E-122	357
Galphao-PA_FBgn0001122	*Px_00831_G-oalpha47A*	88.89	54	6	0	1	54	1	54	2E-16	72
Galphao-PB_FBgn0001122	*Px_00830_G-oalpha47A*	88.10	42	5	0	1	42	1	42	1E-17	75
Galphao-PB_FBgn0001122	*Px_07714_G-oalpha47A*	87.01	154	20	0	1	154	1	154	5E-87	258
Galphao-PB_FBgn0001122	*Px_07716_G-oalpha47A*	98.49	199	3	0	156	354	37	235	3E-149	419
Galphaq-PJ_FBgn0004435	*Px_17406_Galpha49B*	73.11	424	45	2	41	395	305	728	0	618
Galphas-PA_FBgn0001123	*Px_09099_G-salpha60A*	85.08	382	55	1	1	382	34	413	0	652

Table S5. Inventory of JH-related genes in the *Papilio xuthus* genome.

Pathways	Gene name	Gene ID	Gene symbol
Neuropeptide regulation of JH biosynthesis	Allatostatin	*Px_05449_Ast-C*	AS
Neuropeptide regulation of JH biosynthesis	Allatostatin receptor	*Px_11545_AlstR*	ASR
MVA pathway of JH biosynthesis	Acetoacetyl CoA thiolase	*Px_07840_CG10932*	AACT
MVA pathway of JH biosynthesis	Hydroxymethylglutaryl-CoA synthase	*Px_07665_Hmgs*	HMGS
MVA pathway of JH biosynthesis	Hydroxymethylglutaryl-CoA reductase	*Px_14896_Hmgcr*	HMGR
MVA pathway of JH biosynthesis	Mevalonate kinase	*Px_12162_CG33671*	MevK
MVA pathway of JH biosynthesis	Phosphomevalonate kinase	*Px_03089_CG10268*	MevPK
MVA pathway of JH biosynthesis	Diphosphomevalonate decarboxylase	*Px_09639_CG8239*	MevPPD
MVA pathway of JH biosynthesis	Isopentenyl-diphosphate delta-isomerase	*Px_15760_CG5919*	IPPI
MVA pathway of JH biosynthesis	Farnesyl diphosphate synthetase	*Px_02635_Fpps*	FPPS
MVA pathway of JH biosynthesis	Farnesyl diphosphate synthetase-like protein 1	*Px_02636_Fpps*	FPPS-l1
MVA pathway of JH biosynthesis	Farnesyl diphosphate synthetase-like protein 2	*Px_02637_Fpps*	FPPS-l2
MVA pathway of JH biosynthesis	Farnesyl diphosphate synthetase-like protein 3	*Px_02638_Fpps*	FPPS-l3
MVA pathway of JH biosynthesis	Farnesyl diphosphate synthetase-like protein 4	*Px_02639_Fpps*	FPPS-l4
MVA pathway of JH biosynthesis	Farnesyl diphosphate synthetase-like protein 5	*Px_02640_Fpps*	FPPS-l5
MVA pathway of JH biosynthesis	Farnesyl diphosphate synthetase-like protein 6	*Px_02641_Fpps*	FPPS-l6
MVA pathway of JH biosynthesis	Farnesyl diphosphate synthetase-like protein 7	*Px_02642_Fpps*	FPPS-l7
MVA pathway of JH biosynthesis	Farnesyl diphosphate synthetase-like protein 8	*Px_02643_Fpps*	FPPS-l8
MVA pathway of JH biosynthesis	Farnesyl diphosphate synthetase-like protein 9	*Px_09247_Fpps*	FPPS-l9
MVA pathway of JH biosynthesis	Farnesyl diphosphate synthetase-like protein 10	*Px_09248_Fpps*	FPPS-l10
MVA pathway of JH biosynthesis	Farnesyl diphosphate synthetase-like protein 11	*Px_04052_Fpps*	FPPS-l11
MVA pathway of JH biosynthesis	Farnesyl diphosphate synthetase-like protein 12	*Px_00691_Fpps*	FPPS-l12
MVA pathway of JH biosynthesis	Farnesyl diphosphate synthetase-like protein 13	*Px_00692_Fpps*	FPPS-l13
Isoprenoid branch of JH biosynthesis	Farnesyl phosphatase	*Px_10975_CG5567*	FPPase
Isoprenoid branch of JH biosynthesis	Farnesyl phosphatase-like protein 1	*Px_15520_CG15739*	FPPase-l1
Isoprenoid branch of JH biosynthesis	Aldehyde dehydrogenase	*Px_00423_CG8665*	ALDH
Isoprenoid branch of JH biosynthesis	Aldehyde dehydrogenase-like protein 1	*Px_00881_CG31075*	ALDH-l1

Isoprenoid branch of JH biosynthesis	Aldehyde dehydrogenase-like protein 2	*Px_00882_CG31075*	ALDH-l2
Isoprenoid branch of JH biosynthesis	Aldehyde dehydrogenase-like protein 3	*Px_07509_Aldh*	ALDH-l3
Isoprenoid branch of JH biosynthesis	Aldehyde dehydrogenase-like protein 4	*Px_09251_Aldh-III*	ALDH-l4
Isoprenoid branch of JH biosynthesis	Aldehyde dehydrogenase-like protein 5	*Px_13717_CG8665*	ALDH-l5
Isoprenoid branch of JH biosynthesis	Aldehyde dehydrogenase-like protein 6	*Px_16293_CG31075*	ALDH-l6
Isoprenoid branch of JH biosynthesis	Aldehyde dehydrogenase-like protein 7	*Px_16294_CG31075*	ALDH-l7
Isoprenoid branch of JH biosynthesis	Aldehyde dehydrogenase-like protein 8	*Px_13741_CG31075*	ALDH-l8
Isoprenoid branch of JH biosynthesis	Aldehyde dehydrogenase-like protein 9	*Px_17715_CG9629*	ALDH-l9
Isoprenoid branch of JH biosynthesis	Juvenile hormone acid methyltransferase	*Px_16318_jhamt*	JHAMT
Isoprenoid branch of JH biosynthesis	Farnesoic acid O-methyltransferase-like protein 1	*Px_08714_CG10527*	FAMeT-l1
Isoprenoid branch of JH biosynthesis	Farnesoic acid O-methyltransferase-like protein 2	*Px_08715_CG10527*	FAMeT-l2
Isoprenoid branch of JH biosynthesis	Cytochrome P450 15A1	*Px_03301_Cyp304a1*	Cyp15A1
Isoprenoid branch of JH biosynthesis	Cytochrome P450 15C1	*Px_14920_Cyp303a1*	Cyp15C1
JH metabolism	Juvenile hormone esterase	*Px_08363_CG10175*	JHE
JH metabolism	Juvenile hormone esterase-like 1	*Px_14385_CG6018*	JHE-l1
JH metabolism	Juvenile hormone esterase-like 2	*Px_15645_alpha-Est10*	JHE-l2
JH metabolism	Juvenile hormone epoxide hydrolase	*Px_00048_Jheh3*	JHEH
JH metabolism	Juvenile hormone epoxide hydrolase-like protein 1	*Px_00144_Jheh3*	JHEH-l1
JH metabolism	Juvenile hormone epoxide hydrolase-like protein 2	*Px_00150_Jheh3*	JHEH-l2
JH metabolism	Juvenile hormone epoxide hydrolase-like protein 3	*Px_00168_Jheh3*	JHEH-l3
JH metabolism	Juvenile hormone epoxide hydrolase-like protein 4	*Px_00261_Jheh3*	JHEH-l4
JH metabolism	Juvenile hormone epoxide hydrolase-like protein 5	*Px_00866_Jheh1*	JHEH-l5
JH metabolism	Juvenile hormone epoxide hydrolase-like protein 6	*Px_00867_Jheh1*	JHEH-l6
JH metabolism	Juvenile hormone epoxide hydrolase-like protein 7	*Px_01789_Jheh1*	JHEH-l7
JH metabolism	Juvenile hormone epoxide hydrolase-like protein 8	*Px_01868_Jheh2*	JHEH-l8
JH metabolism	Juvenile hormone epoxide hydrolase-like protein 9	*Px_02859_Jheh1*	JHEH-l9
JH metabolism	Juvenile hormone epoxide hydrolase-like protein 10	*Px_05524_Jheh2*	JHEH-l10
JH metabolism	Juvenile hormone epoxide hydrolase-like protein 11	*Px_05525_Jheh2*	JHEH-l11
JH metabolism	Juvenile hormone epoxide hydrolase-like protein 12	*Px_05526_Jheh2*	JHEH-l12
JH metabolism	Juvenile hormone epoxide hydrolase-like protein 13	*Px_05527_Jheh2*	JHEH-l13
JH metabolism	Juvenile hormone epoxide hydrolase-like protein 14	*Px_05528_Jheh2*	JHEH-l14
JH metabolism	Juvenile hormone epoxide hydrolase-like protein 15	*Px_05529_Jheh3*	JHEH-l15
JH metabolism	Juvenile hormone epoxide hydrolase-like protein 16	*Px_05530_Jheh1*	JHEH-l16

JH metabolism	Juvenile hormone diol kinase	*Px_04576_Scp2*	JHDK1
JH metabolism	Juvenile hormone diol kinase	*Px_05680_Scp2*	JHDK2
JH metabolism	Juvenile hormone diol kinase	*Px_05681_Scp2*	JHDK3
JH metabolism	Juvenile hormone diol kinase	*Px_05682_Scp2*	JHDK4
JH metabolism	Juvenile hormone diol kinase	*Px_05683_Scp2*	JHDK5
JH metabolism	Juvenile hormone diol kinase	*Px_05684_Scp2*	JHDK6
JH metabolism	Juvenile hormone diol kinase	*Px_05685_Scp2*	JHDK7
JH metabolism	Juvenile hormone diol kinase	*Px_05686_Scp2*	JHDK8
JH metabolism	Juvenile hormone binding protein	*Px_01877_CG10407*	JHBP
JH metabolism	Juvenile hormone binding protein	*Px_01878_unknow*	JHBP
JH signaling	FKBP39	*Px_08974_FK506-bp2*	FKBP39_1
JH signaling	FKBP39	*Px_09121_FK506-bp2*	FKBP39_2
JH signaling	FKBP39	*Px_08399_shu*	FKBP39_3
JH signaling	FKBP39	*Px_11890_CG14715*	FKBP39_4
JH signaling	FKBP39	*Px_11344_FK506-bp1*	FKBP39_5
JH signaling	FKBP39	*Px_08270_Fkbp13*	FKBP39_6
JH signaling	FKBP39	*Px_15383_shu*	FKBP39_7
JH signaling	Chd64	*Px_16680_Chd64*	Chd64_1
JH signaling	Chd64	*Px_07816_Chd64*	Chd64_2
JH signaling	Methoprene-tolerant/Methoprene-tolerant 1	*Px_08232_gce*	Met/Met1
JH signaling	Germ cell expressed/Methoprene-tolerant 2	*Px_08233_gce*	Gce/Met2
JH signaling	Germ cell expressed/Methoprene-tolerant 3	*Px_08885_gce*	Gce/Met3
JH signaling	Germ cell expressed/Methoprene-tolerant 4	*Px_08886_gce*	Gce/Met4
JH signaling	Ecdysone receptor	*Px_02834_DopEcR*	EcR
JH signaling	Ultraspiracle	*Px_15303_usp*	USP
JH signaling	Kruppel homolog 1	*Px_07131_Kr-h1*	Kr-h1_1
JH signaling	Kruppel homolog 1	*Px_07132_Kr-h1*	Kr-h1_2
