# Supplementary material for: Integrated Analysis of Transcriptome and Proteome to Reveal Pupal Color Switch in Papilio xuthus Butterflies
Source: Front Genet. 2022 Feb 3;12:795115. doi: 10.3389/fgene.2021.795115 (PMC8852814; doi:10.3389/fgene.2021.795115)
Supplement: Supplementary file 2 [file DataSheet3.pdf]

Supplementary Material

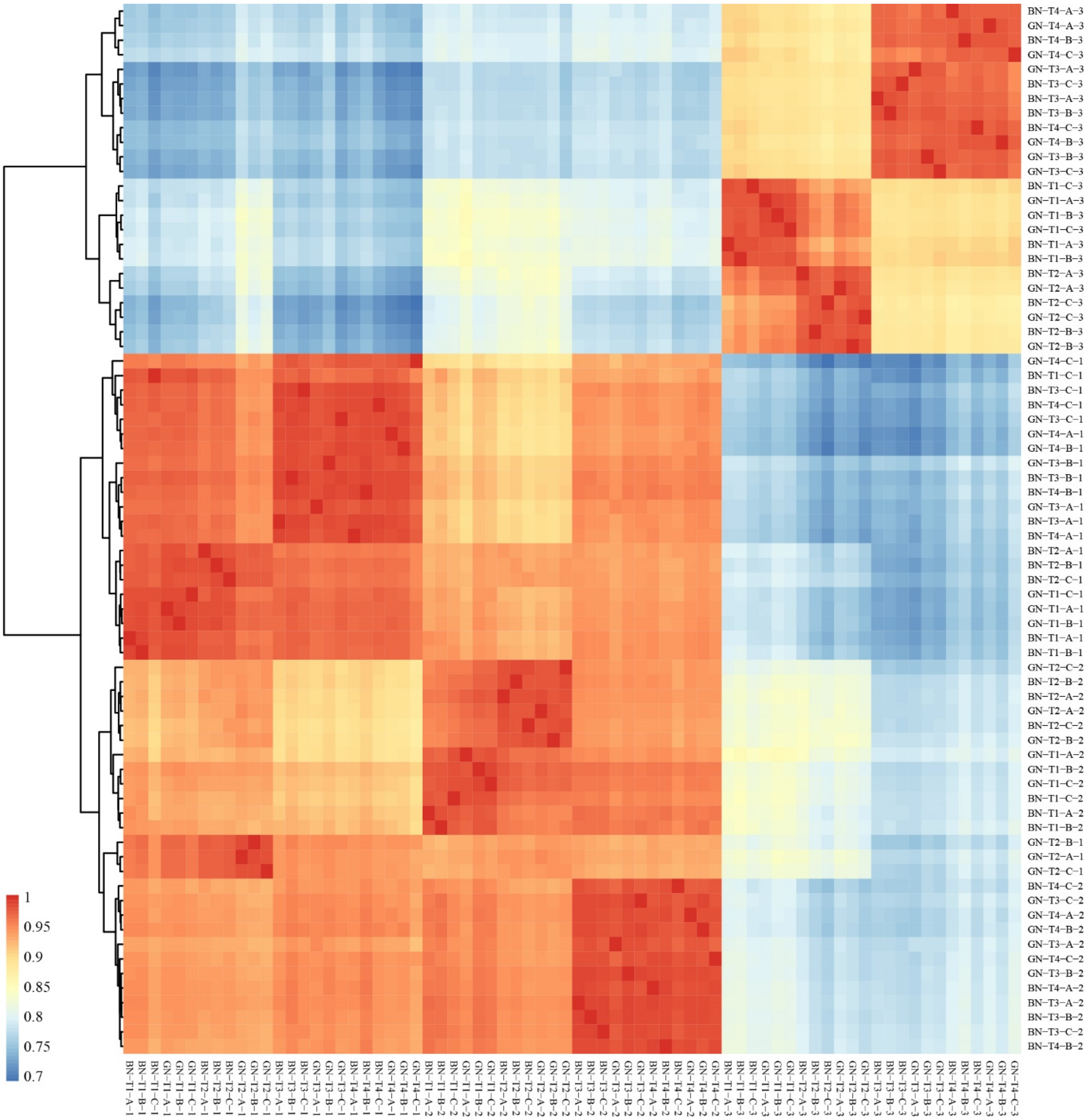

Figure S1. Heatmap of the gene expression profiles in 72 samples including ganglion (-1: Br-SG-TG1 and -2: TG23-AG) and epidermis (-3) of *Papilio xuthus* (Px). Full information of tissues and periods refers to Figure 1 and Table S1.

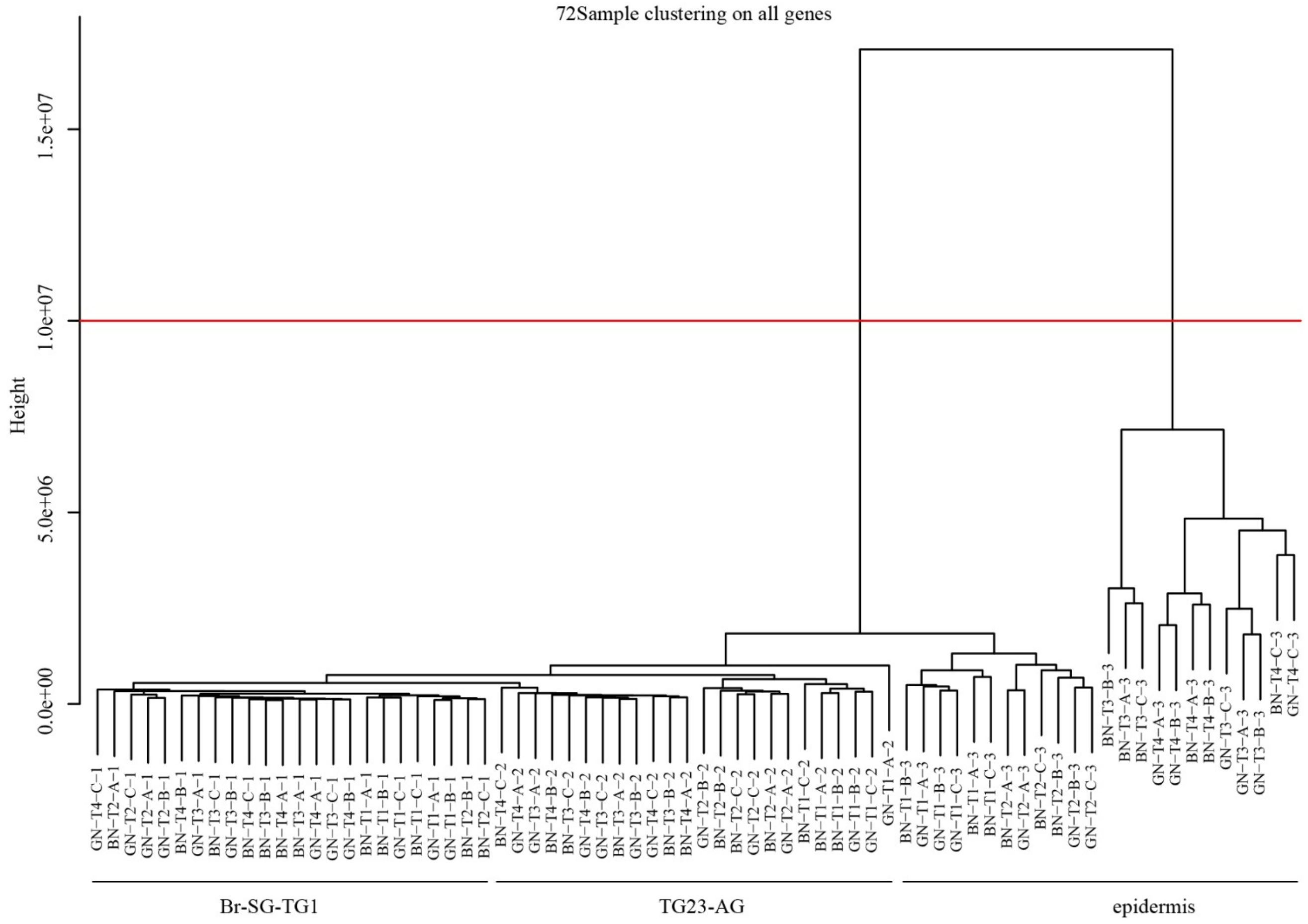

Figure S2. Sample clustering on all genes with average method. The total normalized counts of each gene in 72 samples lower than 2 were excluded.

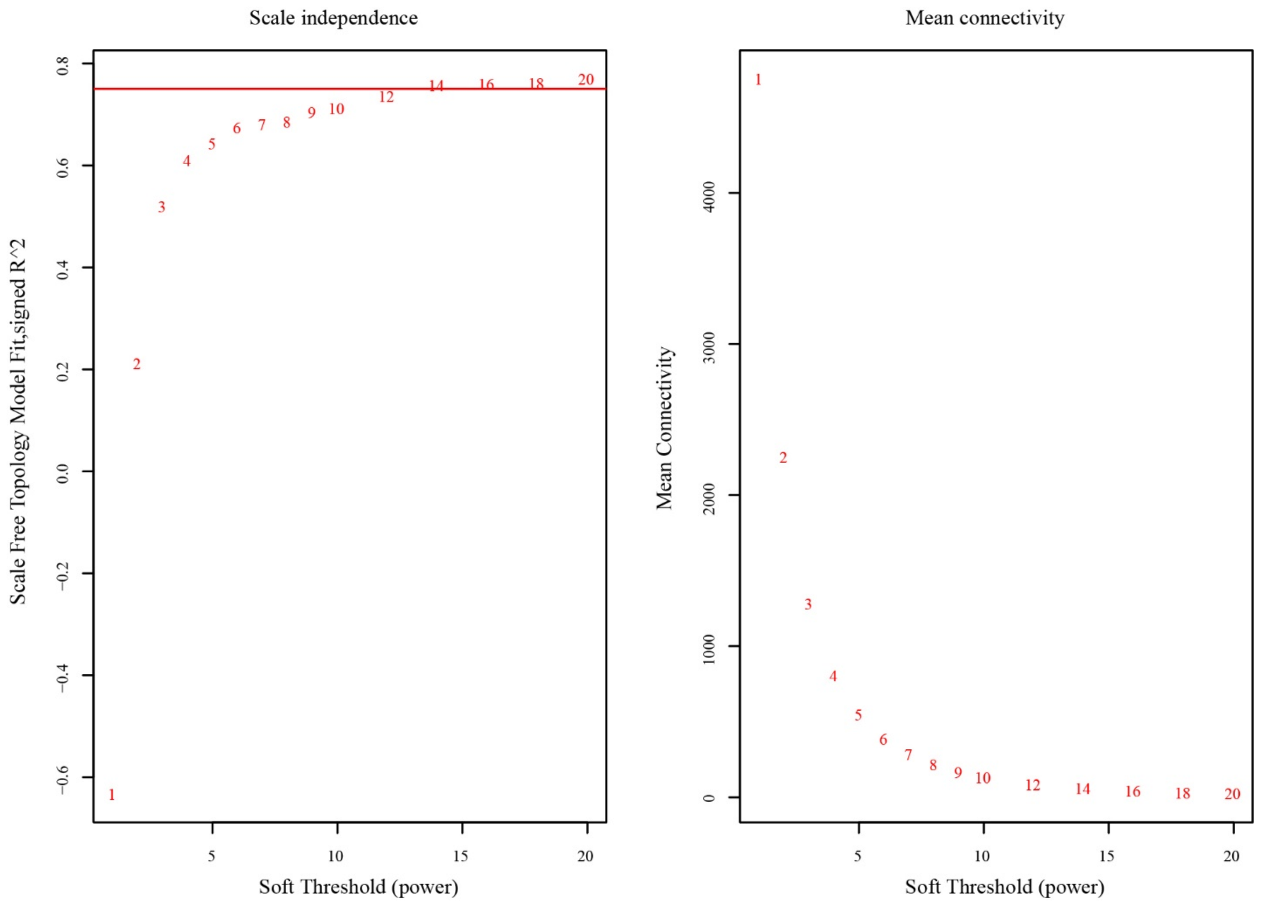

Figure S3. Analysis of network topology for various soft-thresholding powers. The left panel shows the scale-free fit index (y-axis) as a function of the soft-thresholding power (x-axis). The right panel displays the mean connectivity (degree, y-axis) as a function of the soft-thresholding power (x-axis).

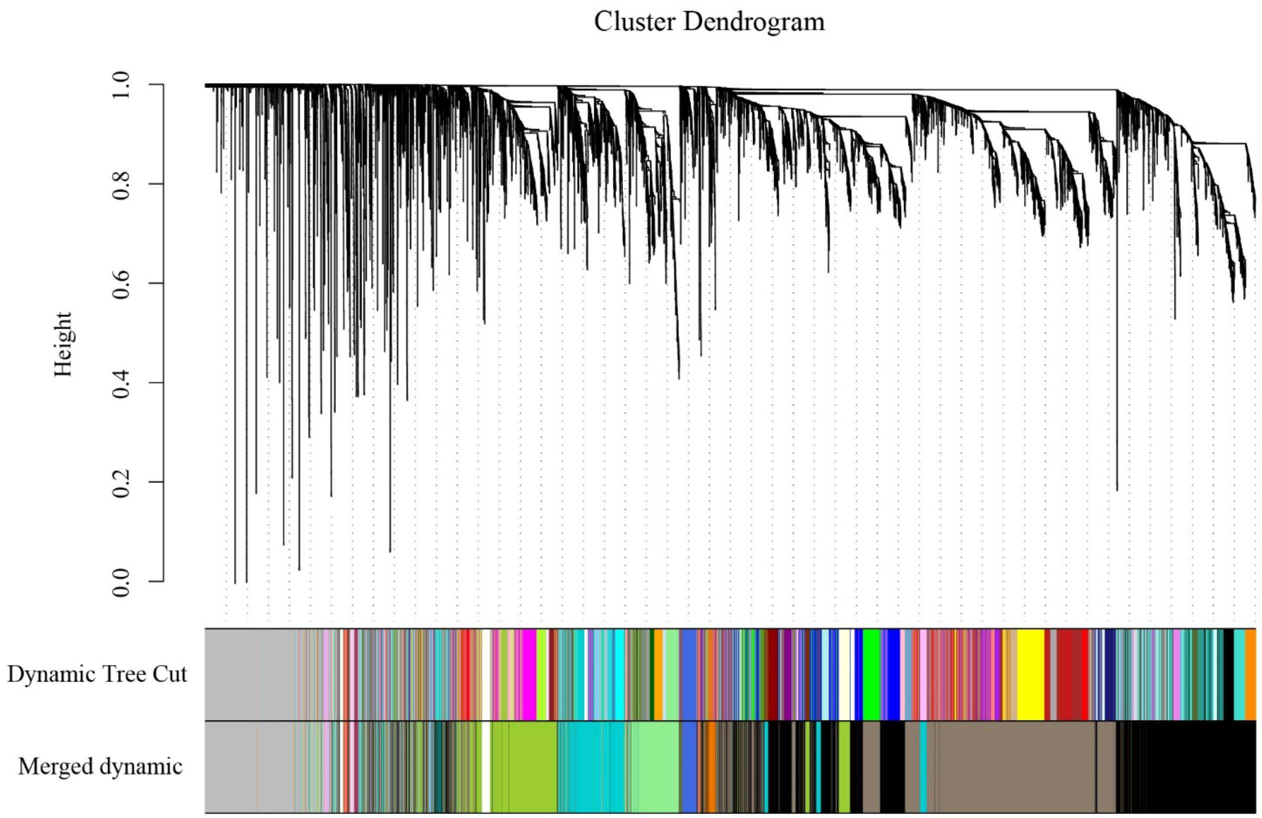

Figure S4. Hierarchical clustering dendrogram of genes, with dissimilarity based on topological overlap, together with assigned module colors.

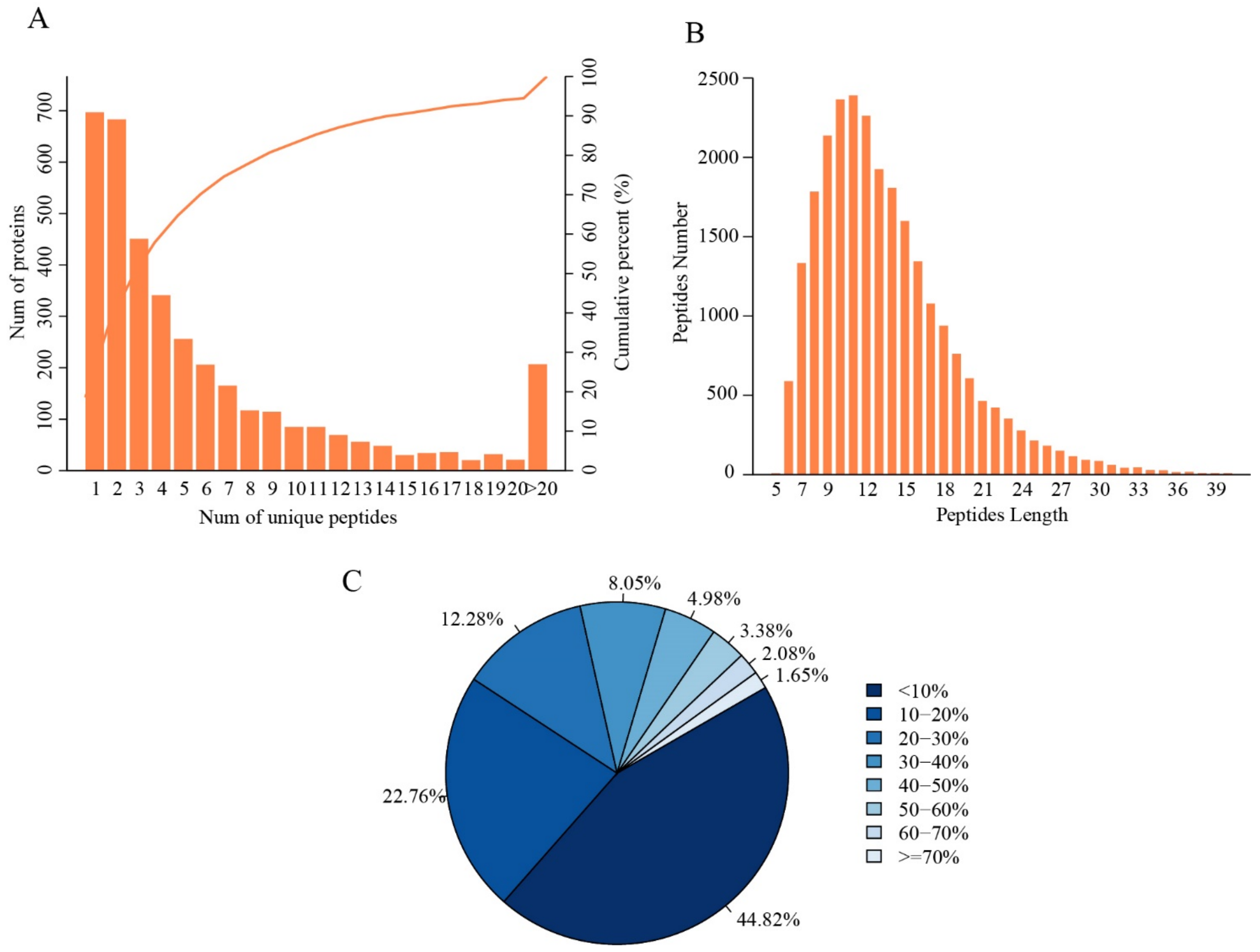

Figure S5. Characteristics of identified proteins. (A) Distribution of unique peptides number. (B) Distribution of peptides length. (C) Distribution of identified protein coverage.

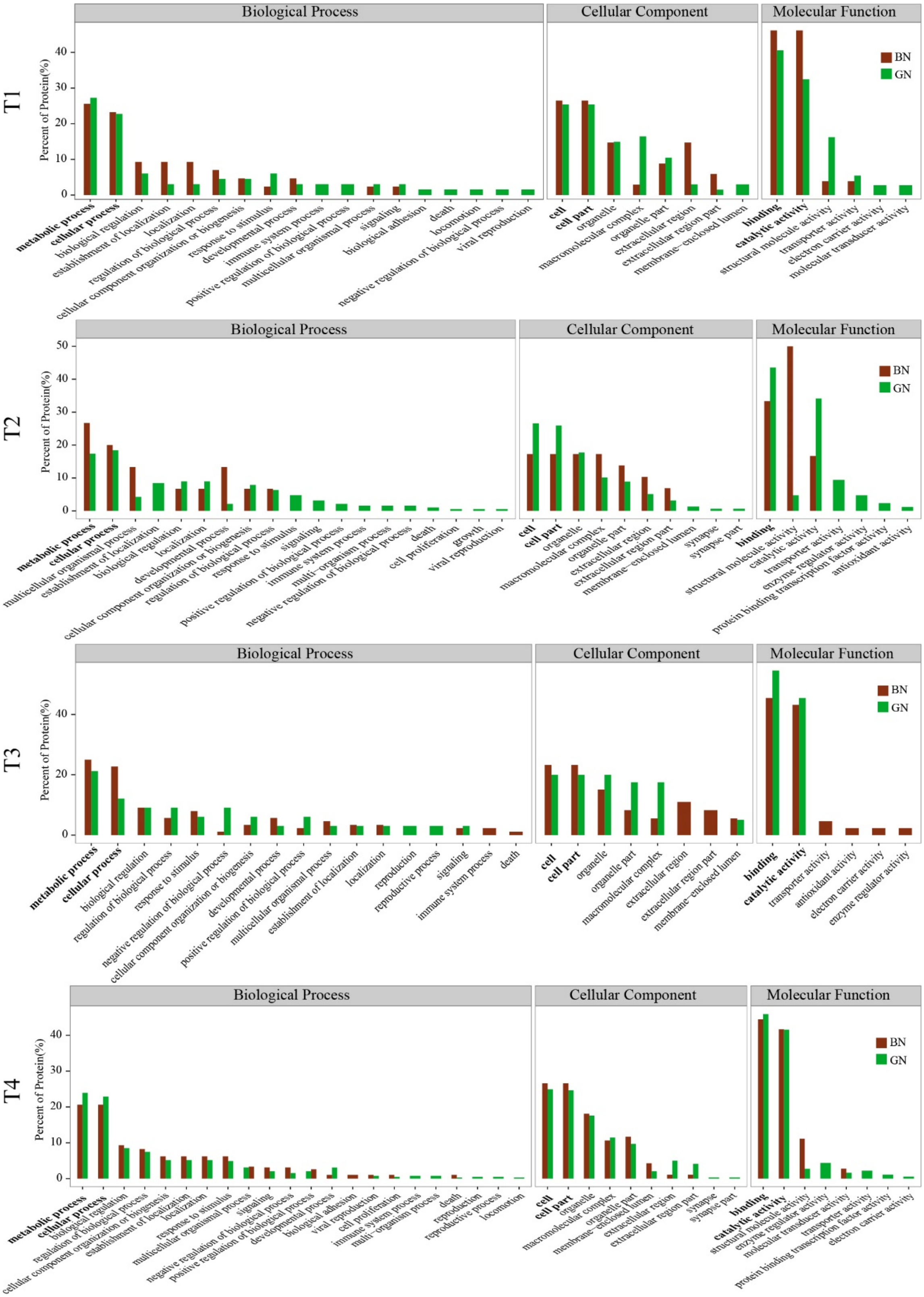

Figure S6. GO enrichment analysis of the identified different abundance proteins (DAPs) at four developmental stages (T1–T4) under green and brown conditions. P-value <0.05. BN and GN represent under brown and green conditions, respectively.

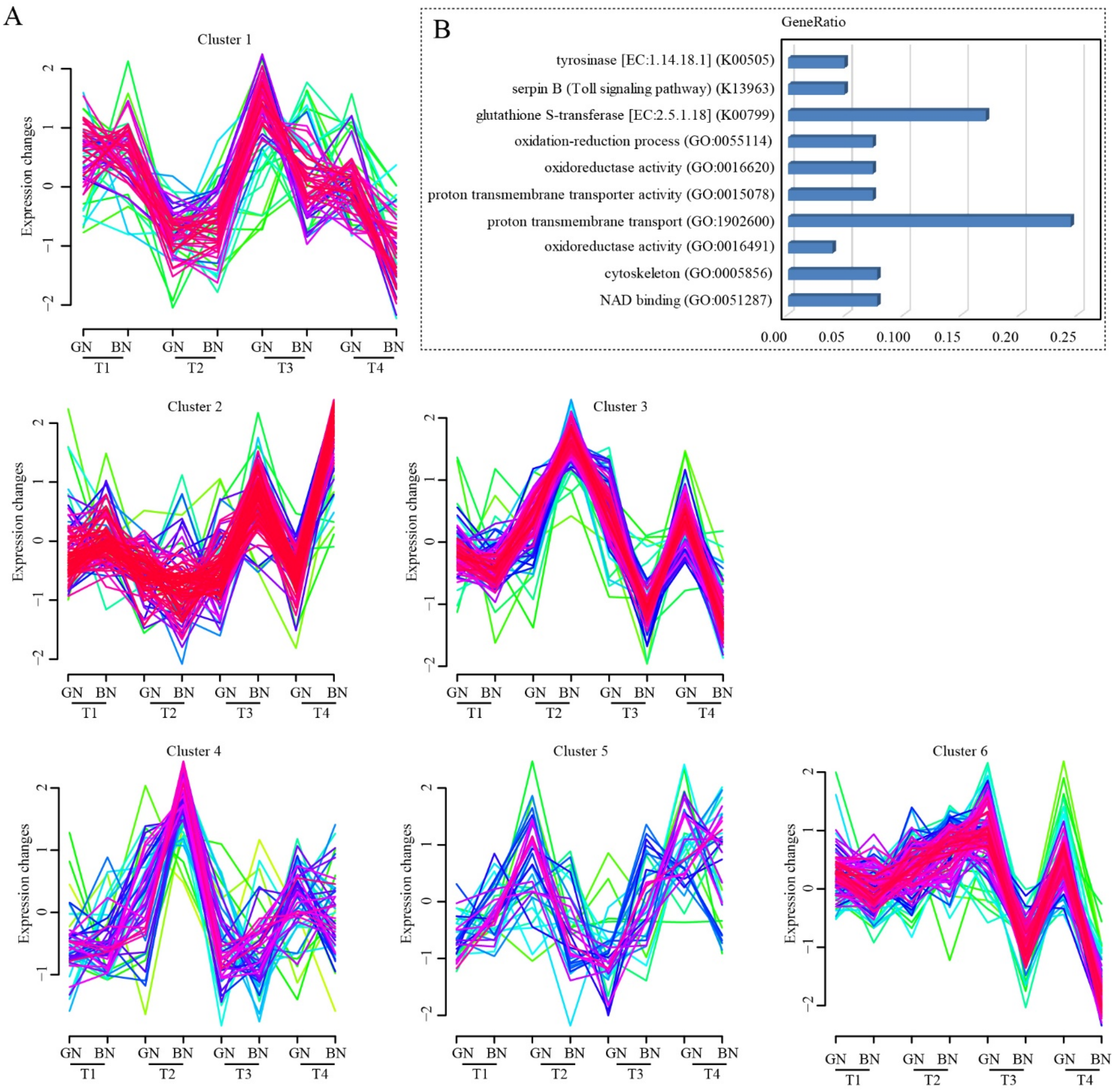

Figure S7. Soft clusters in the expression profiles of different abundance proteins (DAPs) in Mfuzz analysis. (A) The expression profiles of six clusters. Yellow or green colored lines correspond to genes with low membership value. Red and purple lines correspond to genes with high membership value. (B) GO and KEGG enrichment of DAPs in cluster 1.

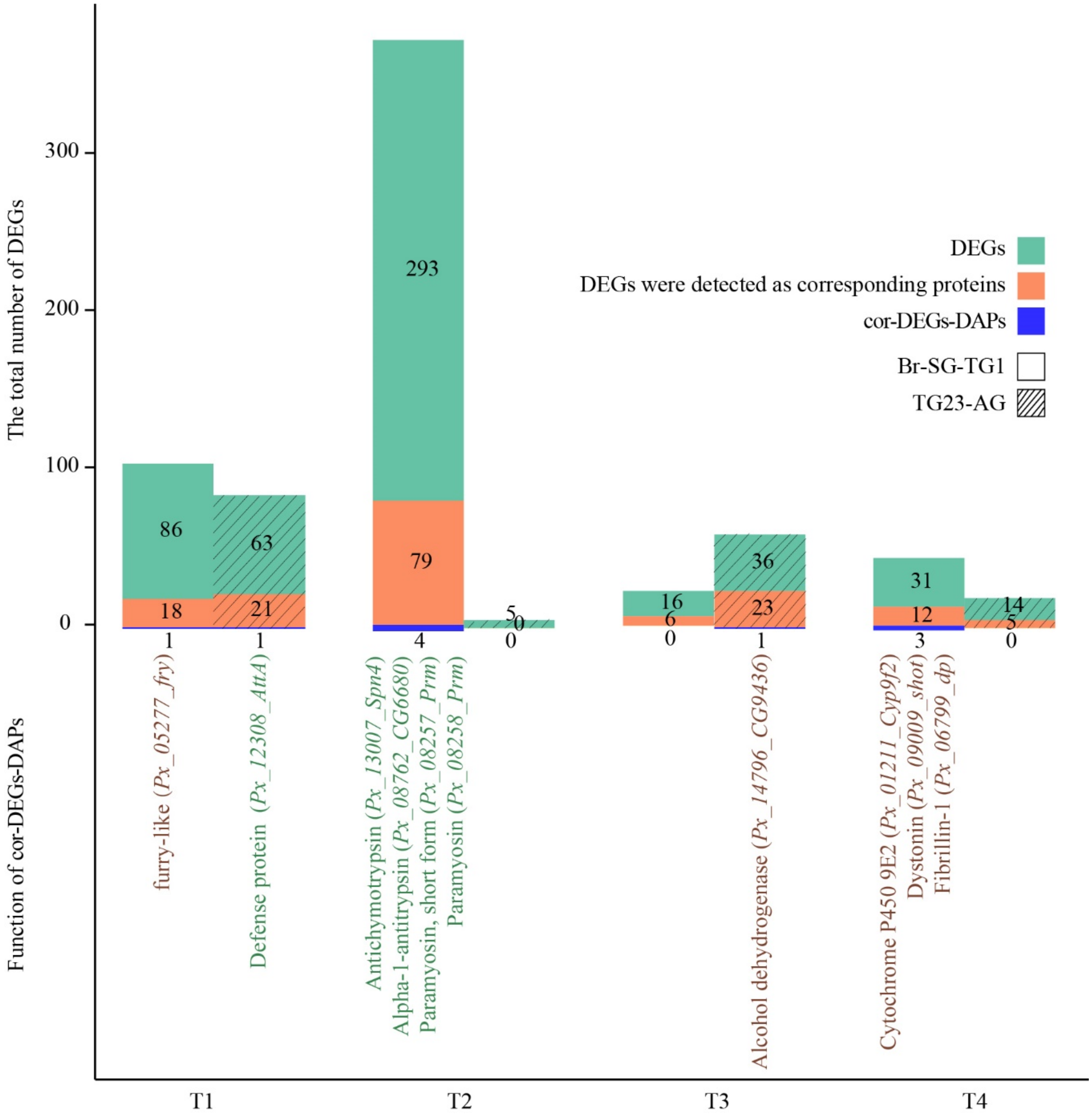

Figure S8. Correlation analysis for gene transcript abundances and their corresponding protein levels. Full information of tissues and periods refers to Figure 1.

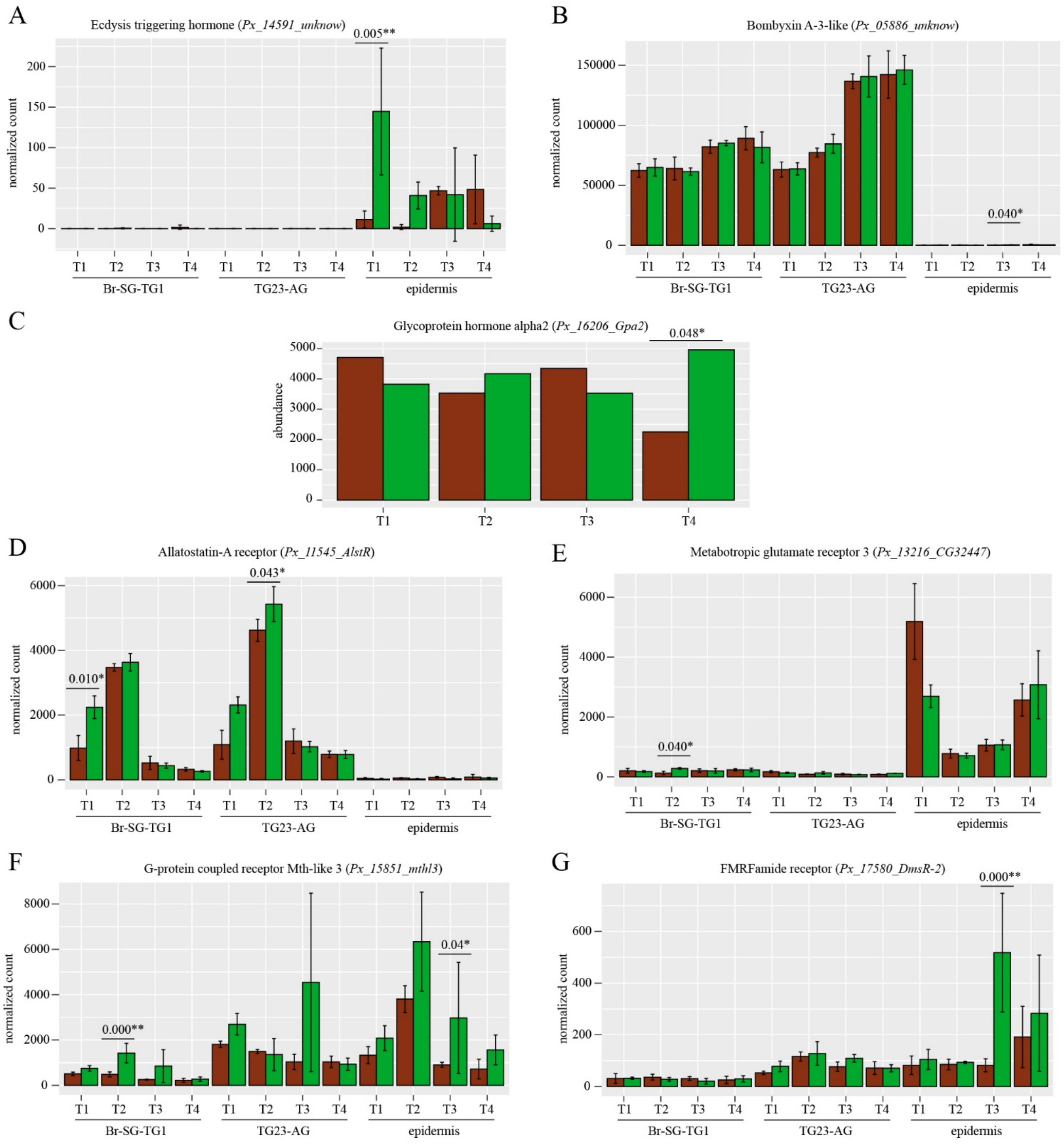

Figure S9. Expression profiles of gene related to neuropeptides and GPCRs under green (GN bar) and brown conditions (BN bar) at transcriptional level or protein level, including neuropeptides (A: ecdysis triggering hormone, B: bombyxin A-3-like, and C: glycoprotein hormone alpha2), GPCRs (D: allatostatin-A receptor, E: metabotropic glutamate receptor 3, F: G-protein coupled receptor Mth-like 3, and G: FMRFamide receptor). Error bars in RNA-seq data indicate SD (n = 3). The number and marker (* <0.05 and ** <0.01) above the line is the adjusted P-value. Full information of tissues and periods refers to Figure 1.

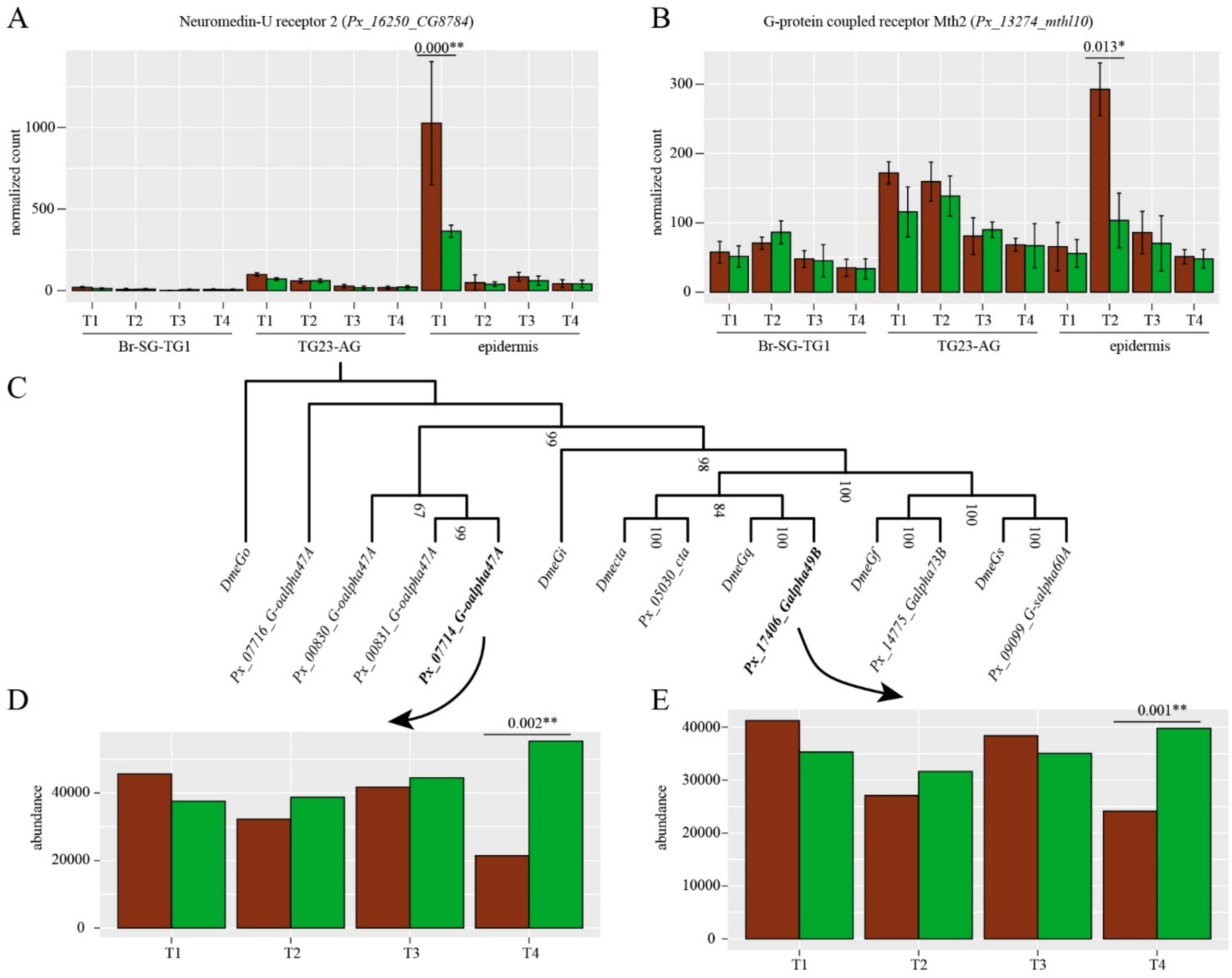

Figure S10. Expression profiles of gene related to GPCRs and G-protein under green (GN bar) and brown conditions (BN bar) at transcriptional level or protein level. (A–B). Expression profiles of two GPCRs (neuromedin-U receptor 2 and G-protein coupled receptor Mth2) at transcriptional level. (C). The maximum likelihood (ML) tree based on G-proteins of *Droshophila melanogaster* and *Papilio xuthus*. (D–E). Expression profiles of two G-proteins (*Px_07714_G-oalpha47A* and *Px_17406_Galpha49B*) at protein level. Error bars in RNA-seq data indicate SD (n = 3). The number and marker (* <0.05 and ** <0.01) above the line is the adjusted P-value. Full information of tissues and periods refers to Figure 1.

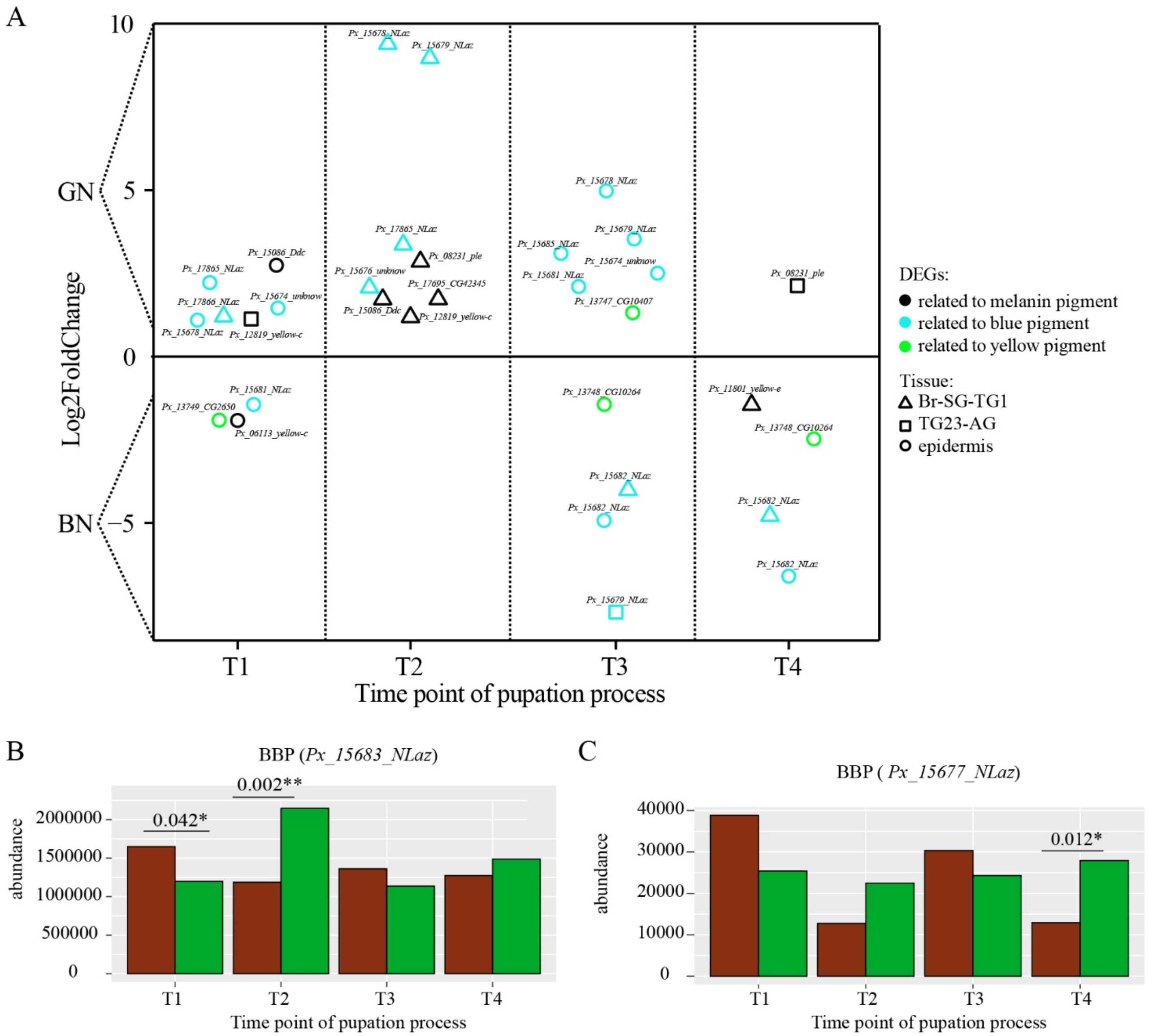

Figure S11. Expression profiles of pigment related genes under green (GN) and brown conditions (BN). (A) The scatter plot of differential expression genes (DEGs) at transcriptional level. (B–C) Expression profiles of two BBPs at protein level. The number and marker (* <0.05 and ** <0.01) above the line is the adjusted P-value. Full information of tissues and periods refers to Figure 1.

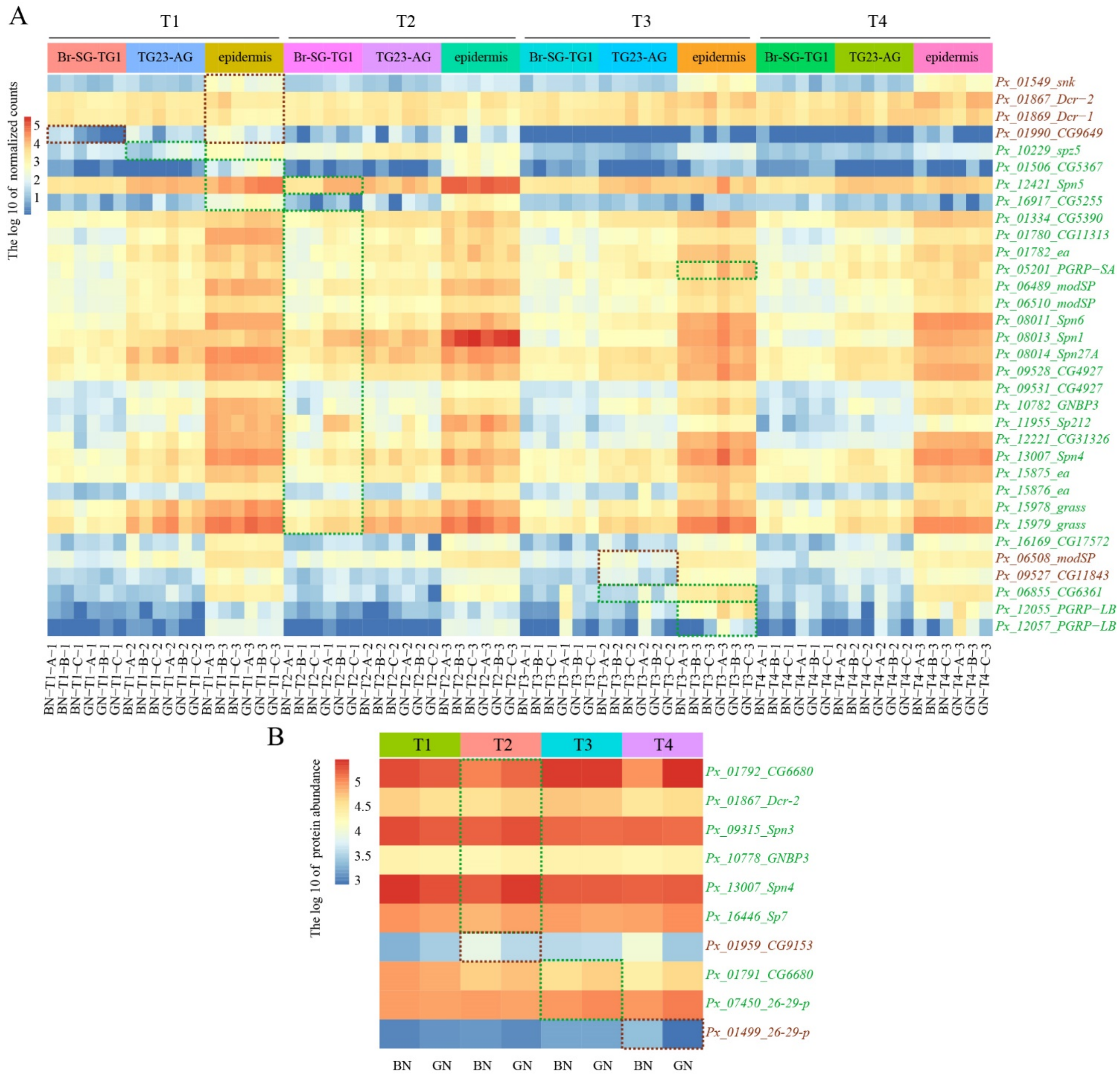

Figure S12. Heatmap of the expression profiles of the Toll signaling pathway related genes at transcriptional level (A) or protein level (B) under green (GN) and brown conditions (BN). The dotted box indicates the specific time-points and tissues of differential expressed genes. Full information of tissues and periods refers to Figure 1.

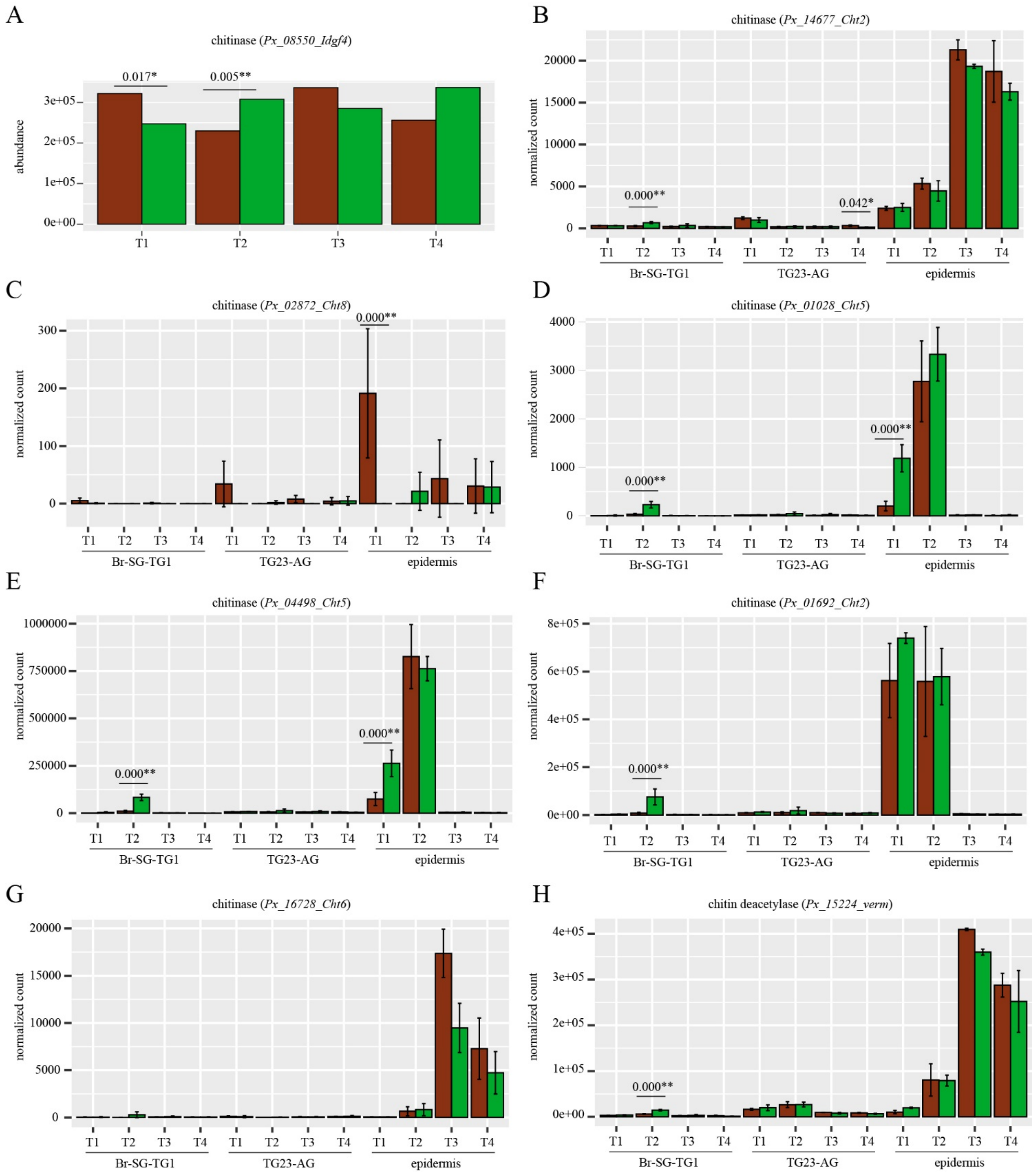

Figure S13. Expression profiles of chitin related genes under green (GN bar) and brown conditions (BN bar) at transcriptional level or protein level, including chitinases (A–G) and chitin deacetylase (H). Error bars in RNA-seq data indicate SD (n = 3). The number and marker (* <0.05 and ** <0.01) above the line is the adjusted P-value. Full information of tissues and periods refers to Figure 1.
